# MicroRNAs of the *mir-17~92* cluster regulate multiple aspects of pancreatic tumor development and progression

**DOI:** 10.18632/oncotarget.16277

**Published:** 2017-03-16

**Authors:** Brian Quattrochi, Anushree Gulvady, David R. Driscoll, Makoto Sano, David S. Klimstra, Christopher E. Turner, Brian C. Lewis

**Affiliations:** ^1^ Department of Molecular, Cell and Cancer Biology, University of Massachusetts Medical School, Worcester, MA 01605, USA; ^2^ Department of Cell and Developmental Biology, State University of New York Upstate Medical Center, Syracuse, NY 13210, USA; ^3^ Division of Pathology, Department of Pathology and Microbiology, Nihon University School of Medicine, Itabashi-ku, Tokyo, 173-8610, Japan; ^4^ Department of Pathology, Memorial Sloan-Kettering Cancer Center, New York, NY 10065, USA; ^5^ Program in Molecular Medicine, University of Massachusetts Medical School, Worcester, MA 01605, USA; ^6^ Department of Radiation Oncology, University of Massachusetts Medical School, Worcester, MA 01605, USA

**Keywords:** pancreatic cancer, mir-17~92, PanIN, regression, invasion

## Abstract

Pancreatic ductal adenocarcinoma (PDAC) is a lethal malignancy characterized by resistance to currently employed chemotherapeutic approaches. Members of the *mir-17~92* cluster of microRNAs (miRNAs) are upregulated in PDAC, but the precise roles of these miRNAs in PDAC are unknown. Using genetically engineered mouse models, we show that loss of *mir-17~92* reduces ERK pathway activation downstream of mutant KRAS and promotes the regression of KRAS^G12D^-driven precursor pancreatic intraepithelial neoplasias (PanINs) and their replacement by normal exocrine tissue. In a PDAC model driven by concomitant KRAS^G12D^ expression and *Trp53* heterozygosity, *mir-17~92* deficiency extended the survival of mice that lacked distant metastasis. Moreover, *mir-17~92*-deficient PDAC cell lines display reduced invasion activity in transwell assays, form fewer invadopodia rosettes than *mir-17~92*-competent cell lines and are less able to degrade extracellular matrix. Specific inhibition of miR-19 family miRNAs with antagomirs recapitulates these phenotypes, suggesting that miR-19 family miRNAs are important mediators of PDAC cell invasion. Together these data demonstrate an oncogenic role for *mir-17~92* at multiple stages of pancreatic tumorigenesis and progression; specifically, they link this miRNA cluster to ERK pathway activation and precursor lesion maintenance *in vivo* and identify a novel role for miR-19 family miRNAs in promoting cancer cell invasion.

## INTRODUCTION

Pancreatic ductal adenocarcinoma (PDAC) is the most common and the deadliest form of pancreatic cancer, comprising 85% of all cases, with a five-year survival rate of just 6.7% [1, 2]. PDAC commonly arises from precancerous lesions called pancreatic intraepithelial neoplasias (PanINs) [3]. These lesions are characterized by very high occurrence of mutations in the *KRAS* oncogene that are also maintained throughout disease progression and found in over 90% of PDAC cases [4]. These findings indicate that KRAS could be a robust therapeutic target in PDAC. Indeed, murine pancreatic cancers with activated *KRAS* (e.g. *KRAS^G12D^* and *KRAS^G12V^*) exhibit oncogene addiction, whereby suppression of *KRAS* activity induces cell death in advanced tumors and regression of early PanINs [5–7]. However, efforts to directly inhibit KRAS activity in human tumors have thus far been unsuccessful [8]. Moreover, clinical and preclinical studies have demonstrated the complexities of inhibiting the well-characterized downstream RAF/MEK/ERK and PI3K/AKT pathways [9–14]. These findings highlight the complexity of the signaling networks downstream of activated KRAS and suggest potential roles for post-transcriptional mechanisms that may buffer signaling downstream of KRAS. Therefore, a deeper understanding of the factors that influence KRAS-driven tumor initiation and progression in the pancreas is greatly needed.

MicroRNAs (miRNAs) are highly conserved short non-coding RNAs that influence gene expression post-transcriptionally and regulate development, normal physiology and disease [15]. MiRNAs have been demonstrated to regulate the initiation and progression of many malignancies by controlling oncogenic and tumor suppressive pathways [16]. Among the earliest described oncogenic miRNAs were members of the *mir-17~92* cluster [17, 18]. *mir-17~92* has been implicated in a variety of cancer contexts [19], and inhibition of members of this cluster has been shown to impair tumor growth and survival [20, 21]. Profiling of human pancreatic tumors and pancreatic cancer cell lines has shown that miRNAs encoded by the *mir-17~92* cluster and its paralogs–*mir-106b~25* and *mir-106a~363*–are upregulated in tumors compared to normal pancreatic tissue or chronic pancreatitis [22, 23]. In addition, *mir-17~92*-encoded miRNAs are induced in precursor PanIN lesions, implicating them in early stages of PDAC development [24], and miR-17 overexpression has been associated with reduced pancreatic cancer patient survival [25]. The *mir-17~92* cluster was initially identified as oncogenic over a decade ago [18, 26]. Subsequent studies have demonstrated critical roles for this microRNA cluster in several malignancies including B-cell lymphoma, retinoblastoma, medulloblastoma, hepatocellular carcinoma and neuroblastoma [20, 21, 27–34]. Individual miRNAs within the cluster have been associated with specific tumorigenic properties. Of note, the miR-19 microRNAs have been associated with tumor cell invasion and metastasis in gastric cancer [35], lung cancer [36] and colon cancer [37].

Studies in pancreatic cancer cell lines additionally demonstrated roles for the *mir-17~92* cluster in PDAC cell proliferation, transformation and invasion [25, 38, 39]. However, to date no studies have been performed to evaluate the role of the cluster *in vivo* during pancreatic tumor initiation and progression. Given the upregulation of these miRNAs in human pancreatic cancers and their validated role as oncogenes in a variety of contexts, we hypothesized that they contribute to KRAS-induced pancreatic tumorigenesis. Therefore, we experimentally tested the requirement for *mir-17~92* in a mouse model of pancreatic cancer.

We find that deletion of *mir-17~92* impairs MEK/ERK signaling in PanIN lesions and this correlates with the presence of fewer PanINs, as well as their regression over time. In addition, we find that *mir-17~92* miRNAs, in particular miR-19 family miRNAs, promote PDAC cell invasion by regulating the formation of extracellular matrix-degrading invadopodia rosettes. Together, these findings illustrate important roles for *mir-17~92* miRNAs during multiple phases of PDAC development and progression.

## RESULTS

### *mir-17~92* loss does not impact normal pancreas development

Prior miRNA expression profiling studies of human PDAC specimens demonstrated elevated expression of components of the *mir-17~92* cluster in PDAC. However, the results from these studies were somewhat inconsistent, potentially reflecting the significant stromal and immune cell component of pancreatic tumors. To ascertain whether *mir-17~92* miRNAs have elevated expression in PDAC cells, we profiled a panel of PDAC cell lines as well as the immortalized pancreatic epithelial cell line HPNE. We find that *mir-17~92* miRNAs are consistently overexpressed in PDAC cell lines (Supplementary Figure 1). Thus, we set out to identify the role of this microRNA cluster in pancreatic tumorigenesis *in vivo* in genetically engineered mouse models.

To determine the effect of *mir-17~92* loss on pancreatic development, we induced pancreas-specific deletion of the *mir-17~92* cluster using the conditional *mir-17~92^flox^* allele and the recombination driver *Ptf1a-Cre* [40, 41]. qRT-PCR of RNA from whole pancreata showed a strong reduction in the levels of *mir-17~92* constituent miRNAs in compound *mir-17~92^flox/flox^, Ptf1a-*Cre mice (Supplementary Figure 2A). The observed residual *mir-17~92* expression is likely from endocrine cells, many of which are derived from a PTF1A-independent lineage [40, 42], as well as a small population of acinar and ductal cells that have avoided recombination due to the fact that *Cre* drivers are not 100% efficient [43]. Expression from the paralogous *mir-106b~25* cluster is unaffected (Supplementary Figure 2A). Despite efficient depletion of *mir-17~92* miRNAs from the pancreas, organ size and exocrine and endocrine architecture and composition were unperturbed (Supplementary Figure 2B). These findings demonstrate that *mir-17~92* is not required for normal pancreas development.

### *mir-17~92* loss promotes PanIN loss and exocrine recovery

**Figure 1:**
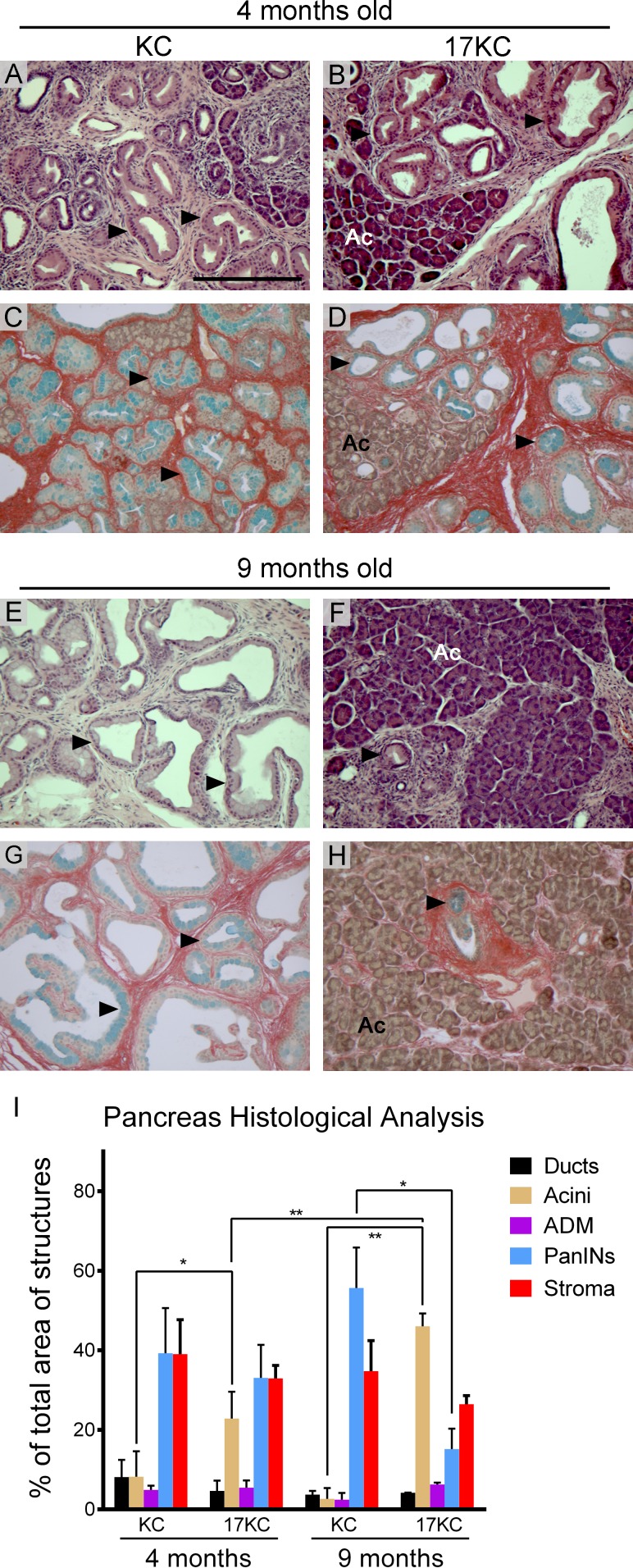
*mir-17~92* null PanINs regress with age Histological evaluation of PanIN lesions in pancreata from 4-month old (**A**–**D**) and 9-month old (**E-H**) KC and 17KC mice. Representative images from hematoxylin and eosin (**A, B**, **E**, **F**) and quadchrome (simultaneous Alcian Blue and Sirius Red staining; **C, D**, **G**, **H**) stains are shown. (**I**) Quantification of normal and neoplastic cell types as a percentage of total tissue area. The quadchrome stain marks collagen red, mucin blue, cytoplasm yellow-brown, and nucleic acids black. Arrowheads identify examples of PanIN lesions. Ac = Acinar tissue. Number of organs (n) analyzed for each group were 4 mo KC (4), 4 mo 17KC (7), 9 mo KC (3), 9 mo 17KC (2). Scale bar = 0.25 mm. *p* values by Student's *t* test: * < 0.05, ** < 0.01. Error bars represent standard deviation from the mean.

To assess the impact of *mir-17~92* deletion on the development and progression of precursor precancerous PanIN lesions, we crossed *mir-17~92^flox/flox^, Ptf1a-Cre* mice onto the *LSL-Kras^G12D^* background [44]. Breeding pairs were designed to cross *mir-17~92^flox/wt^, LSL-Kras^G12D^* mice with *mir-17~92^flox/wt^, Ptf1a-Cre* mice in order to generate littermate *mir-17~92^wt/wt^, LSL-Kras^G12D^, Ptf1a-Cre* and *mir-17~92^flox/flox^, LSL-Kras^G12D^, Ptf1a-Cre* mice (hereafter ‘KC’ and ‘17KC’). Littermate KC and 17KC animals were maintained until four or nine months of age and subsequently euthanized to obtain pancreata for histological analysis. At four months of age, the tissue area occupied by PanIN lesions was not significantly different between the two groups as illustrated by hematoxylin and eosin staining and quadchrome staining (Figure 1A–1D, 1I). However, the extent of normal acinar tissue was significantly greater in the pancreata of 17KC mice (Figure 1I). Nine month-old KC mice displayed higher PanIN burdens compared to younger KC mice and nine month-old 17KC littermates (Figure 1E–1I). In contrast to the trend observed in KC mice, we observed that the pancreata of nine month-old 17KC mice exhibited less PanIN tissue and more healthy acinar tissue by area than four month-old 17KC mice (Figure 1F, 1H, 1I). Of note, PanIN lesions in KC and 17KC pancreata demonstrate no significant differences in proliferation or apoptosis markers and no differences were observed in the proliferative rate of adjacent acinar tissue (Supplementary Figure 3). Together, these data suggest that loss of *mir-17~92* impairs the maintenance of PanIN lesions.

**Figure 2:**
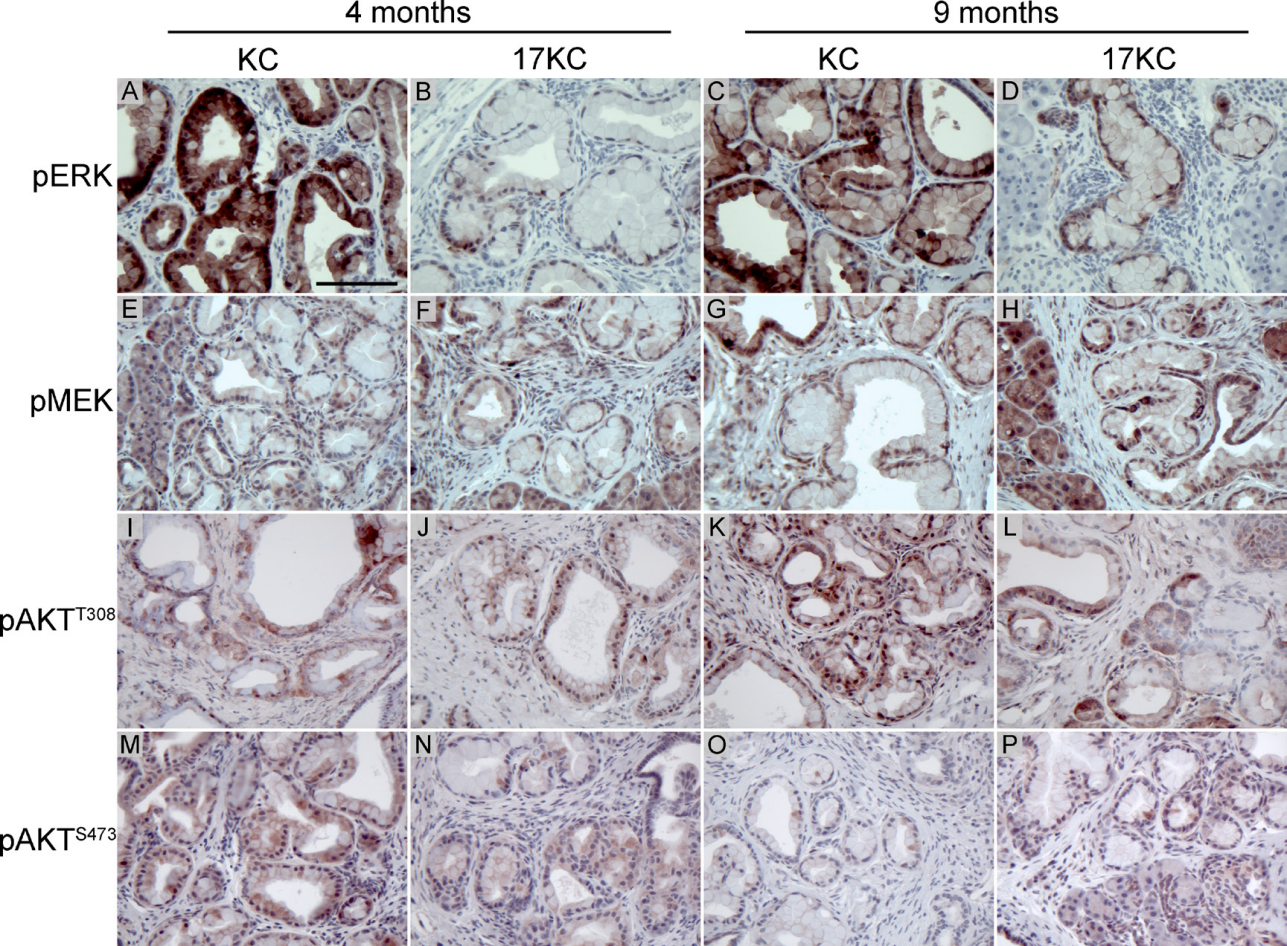
*mir-17~92* null PanINs display reduced MEK/ERK pathway activation Immunostaining of PanIN lesions identified in 4- and 9-month old KC and 17KC mice for phosphorylated ERK (**A**–**D**), phosphorylated MEK (**E**–**H**), AKT phosphorylated at Thr^308^ (**I**–**L**), and AKT phosphorylated at Ser^473^ (**M**–**P**). Scale bar = 0.1 mm.

**Figure 3:**
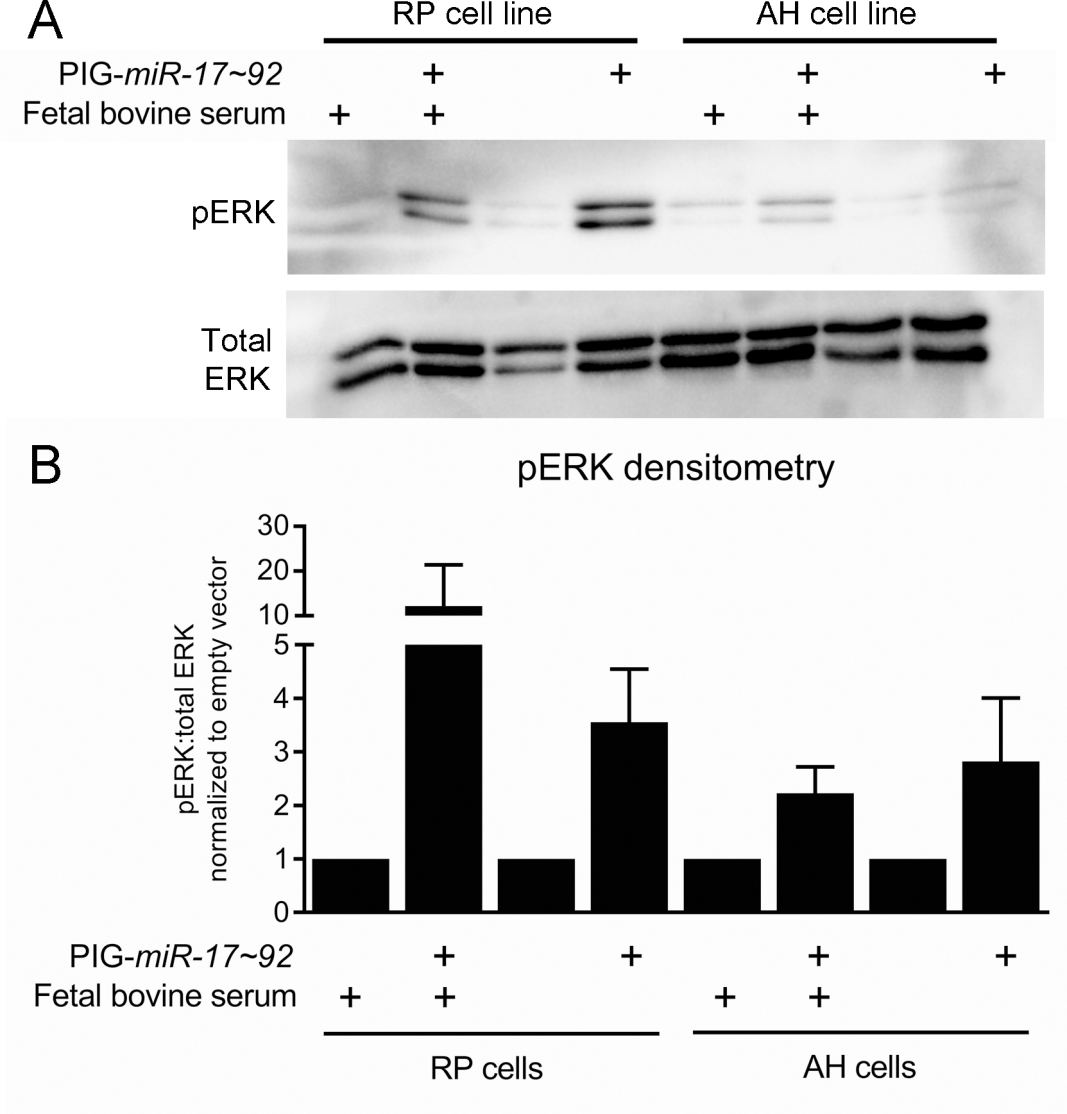
ERK signaling is increased by *mir-17~92* overexpression in PanIN cell lines (**A**) Representative immunoblot for pERK and total ERK in the PanIN cell lines RP2294 and AH2375 stably infected with PIG-*mir-17~92* or empty vector. (**B**) Average densitometry for three experiments performed in (A). Error bars represent standard deviation from the mean.

Recent work suggested that inhibition of MEK/ERK signaling promotes the regression of PanIN lesions [45]. As determined by immunohistochemical staining for phosphorylated ERK, we observed that 17KC PanINs display marked reduction in MAPK signaling (Figure 2A–2D). In contrast to the difference in phosphorylated ERK, phosphorylation of the upstream kinase MEK was not different between the two genotypes, suggesting that signaling through the MEK/ERK cascade is impacted at the level of ERK but not further upstream (Figure 2E–2H). Moreover, ectopic *mir-17~92* expression in murine PanIN cell lines [46, 47] increased p-ERK levels under both serum replete and serum starved conditions (Figure 3A, 3B), confirming that *mir-17~92*-encoded miRNAs regulate this pathway.

Analysis of the phosphorylation status of AKT at the Thr^308^ and Ser^473^ sites that are phosphorylated by PDK1 and mTORC2 also did not show any differences between genotypes (Figure 2I–2P). Taken together with the phosphorylation of MEK, these data suggest that the alterations in ERK phosphorylation do not reflect an overall reduction in KRAS signaling, and further suggest that *mir-17~92* regulates PanIN maintenance by specifically influencing ERK pathway activity downstream of KRAS^G12D^ and MEK.

We utilized the TargetScan database [48] to identify known and putative mRNA targets of *mir-17~92* that are implicated in the regulation of ERK phosphorylation. We identified the dual specificity phosphatases DUSP2, DUSP7 and DUSP10, which are known to regulate MAP kinase phosphorylation, as potential targets of cluster-encoded miRNAs [49, 50]. However, immunostaining of PanIN lesions with antibodies against these phosphatases did not show any differences between *mir-17~92* wild type and deficient PanINs (data not shown). Moreover, the enhanced ERK phosphorylation observed in PanIN cell lines following ectopic *mir-17~92* expression was not associated with changes in DUSP7 or DUSP10 protein levels (Supplementary Figure 4). DUSP2 was undetectable by immunoblotting or qRT-PCR in these cell lines (data not shown). Thus, the detailed mechanisms regulating ERK phosphorylation in PanINs downstream of *mir-17~92* remain unknown.

### *mir-17~92*-deficient tumors display a delayed invasion phenotype

**Figure 4:**
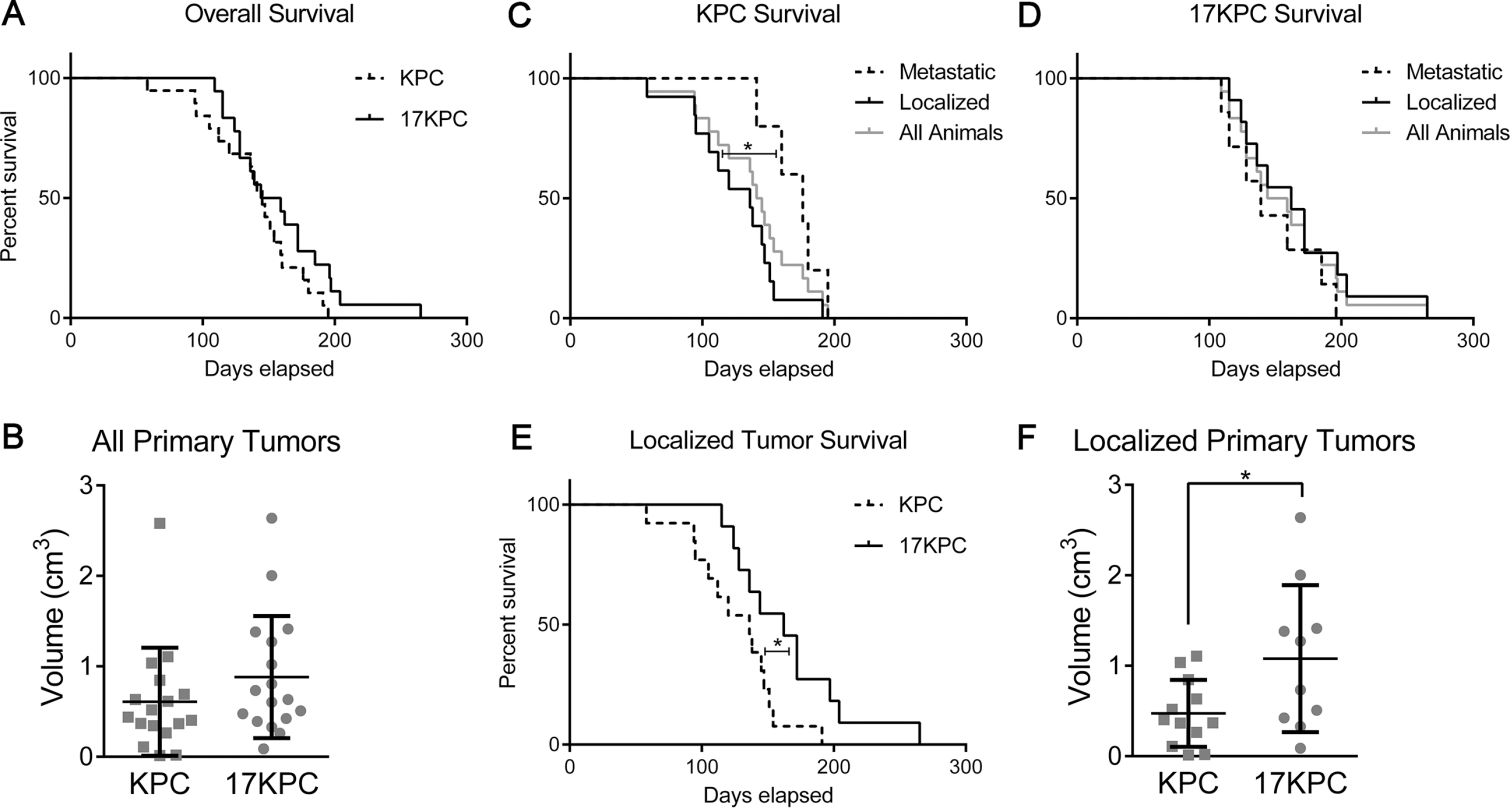
Loss of *mir-17~92* prolongs survival in mice without metastases (**A**) Kaplan-Meier survival plot for KPC and 17KPC mice. (**B**) Primary tumor burden identified in these mice upon euthanasia. (**C**) Kaplan-Meier survival plot comparing metastatic and localized KPC mice. (**D**) Kaplan-Meier survival plot comparing metastatic and localized 17KPC mice. (**E**) Kaplan-Meier survival plot comparing localized KPC and localized 17KPC mice. (**F**) Total primary tumor burden of localized mice. Number of animals (n) for each group were as follows: KPC (18), 17KPC (18), metastatic/localized KPC (5/13), metastatic/localized 17KPC (7/11), female/male KPC (7/11), female/male 17KPC (10/8). *p* values: * < 0.05. Error bars represent standard deviation from the mean.

To assess whether *mir-17~92* deletion impairs progression to carcinoma, we accelerated the KC model by including conditional loss of one copy of *Trp53* (*LSL-Kras^G12D^, Trp53^flox/wt^, Ptf1a-Cre* and *mir-17~92^flox/flox^, LSL-Kras^G12D^, Trp53^flox/wt^, Ptf1a-Cre*; hereafter “KPC” and “17KPC” mice). We observed that KPC and 17KPC mice display similar overall survival and tumor size (Figure 4A, 4B). Rates of liver metastasis are also equivalent between groups (Supplementary Figure 5A, 5B). The carcinomas identified in mice of both genotypes displayed a mixture of glandular and undifferentiated histology; no difference in the relative frequencies of the histologic types was identified between the two groups (Supplementary Figure 5C–5E). Histological evidence of invasion was also equally prevalent in both groups, and variously involved the stomach/duodenum, liver, colon, and spleen (Supplementary Figure 5F–5J). The histopathology findings are summarized in Supplementary Table 1. Additionally, tumors across both groups exhibited similar rates of proliferation and apoptosis as demonstrated by Ki67 and cleaved caspase 3 (CC3) staining (Supplementary Figure 6). Moreover, KPC and 17KPC tumors display equivalent MEK/ERK pathway activation as measured by immunostaining for phosphorylated ERK (Supplementary Figure 7A, 7B). Further, cell lines derived from KPC and 17KPC tumors display similar levels of ERK phosphorylation and similar levels of DUSP7 and DUSP10 expression (Supplementary Figure 7C, 7D). Together, these findings suggest that heterozygous deletion of *Trp53* compensates for the loss of *mir-17~92* and promotes disease progression in *mir-17~92* deficient animals.

In the KPC mouse model, mice reliably generate aggressive primary tumors that invade adjacent tissues and sporadically metastasize: thus, morbidity and euthanasia result from the effects of either the primary tumor or its metastases. To ascertain whether *mir-17~92* deletion differentially impacted primary versus metastatic disease processes, we stratified survival data based on the presence or absence of grossly visible metastases at euthanasia. The majority of KPC mice lacked metastases and were sacrificed at relatively young ages due to effects of the primary tumor (“localized” mice), whereas a minority presented later with metastatic disease (“metastatic mice”) (Figure 4C), suggesting that primary KPC tumors cause significant morbidity prior to the development of metastases. However, this was not observed in 17KPC mice, where localized and metastatic mice display similar survival curves (Figure 4D). Indeed, the survival of metastatic mice is not significantly different between KPC and 17KPC mice (data not shown), but the survival of localized KPC mice was significantly worse than that of localized 17KPC mice (Figure 4E). Moreover, localized 17KPC mice also possessed larger tumors than localized KPC mice (Figure 4F). Together, these data suggest that *mir-17~92* contributes to the morbidity and mortality caused by primary KPC tumors and does not impact time to metastasis.

The observed differences in survival among animals with localized disease could be the result of reduced or delayed invasive potential of 17KPC primary tumors. Thorough histological step sectioning of all primary tumors and adjacent tissues obtained in the study demonstrated equivalent evidence of invasion between KPC and 17KPC animals at the time of euthanasia (data not shown). This suggests that local invasion is a common endpoint in our survival study, but requires more time to develop in 17KPC mice, resulting in the longer survival and larger tumors of localized 17KPC mice compared to localized KPC mice. Localized pancreatic cancer in mice can cause morbidity with biliary obstruction and jaundice (evident in the ears, footpads, or pancreas) or gastrointestinal (GI) obstruction, as seen by gross abdominal distension and GI lumen distension upstream of an adhesion with the absence of downstream luminal contents on necropsy. While these features were commonly observed in KPC mice, 17KPC mice never presented with any form of GI or biliary obstruction (Supplementary Figure 8). Other characteristics of tumor presentation such as the presence of adhesions to adjacent organs, intraperitoneal bleeding, jaundice or ascites demonstrated no significant differences between KPC and 17KPC mice (Supplementary Figure 8). Together, these *in vivo* findings suggest that loss of the *mir-17~92* cluster may impact PDAC cell invasion, a feature associated with later stages of disease.

### *mir-17~92*-deficient cell lines are less invasive *in vitro*

**Figure 5:**
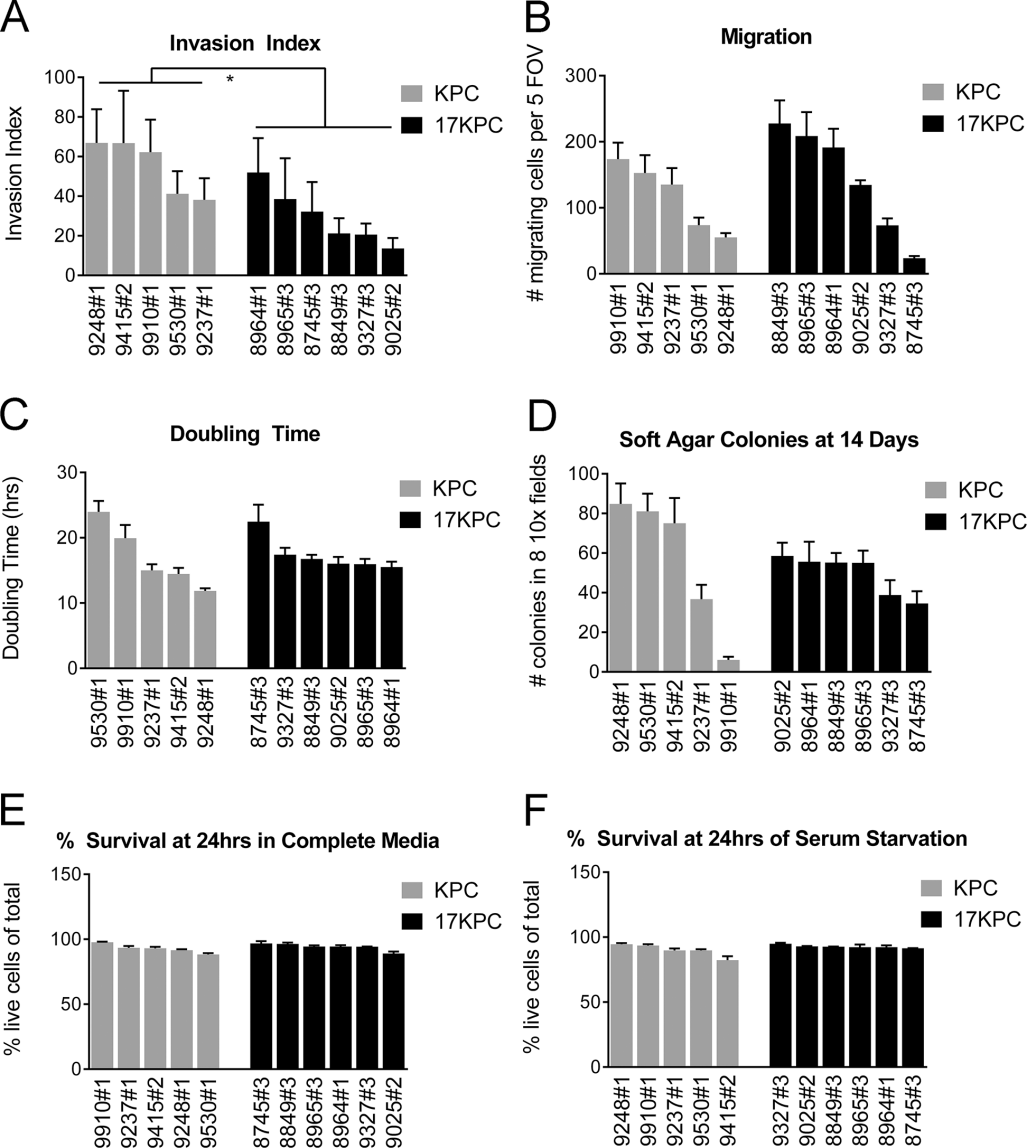
*mir-17~92* null PDAC cell lines have reduced invasive capacity *in vitro* Characterization of *in vitro* phenotypes of PDAC cell lines derived from KPC and 17KPC tumors. (**A**) Invasion activity in transwell assays, displayed as invasion index [(number of invading cells/number of migrating cells) × 100]. (**B**) Migration activity in transwell assays. (**C**) Proliferation rate. (**D**) Colony formation in a soft agar. (**E**) Cell survival in serum-replete medium. (**F**) Cell survival in serum-free medium. Cell line nomenclature is noted as cage#animal# (e.g. 9025#2 is the cell line derived from the primary tumor of mouse #2 from cage #9025). All error bars represent SD. *p value*: * < 0.05.

To better understand the biology of *mir-17~92* deficient pancreatic cancer cells, we generated a collection of cell lines from KPC and 17KPC tumors. Evaluation of cell invasion in transwell assays demonstrated that 17KPC cell lines have reduced ability to invade through Matrigel relative to KPC cell lines (Figure 5A). However, no differences were observed between the two genotypes in their ability to migrate across uncoated membranes (Figure 5B). Additionally, there were no significant differences in the proliferation, anchorage independent growth, or survival phenotypes of KPC and 17KPC cell lines (Figure 5C–5F). These data agree with the suggestion that 17KPC tumors display delayed invasion *in vivo*, and suggest a specific defect in the ability of tumor cells to manipulate extracellular matrix.

**Figure 6:**
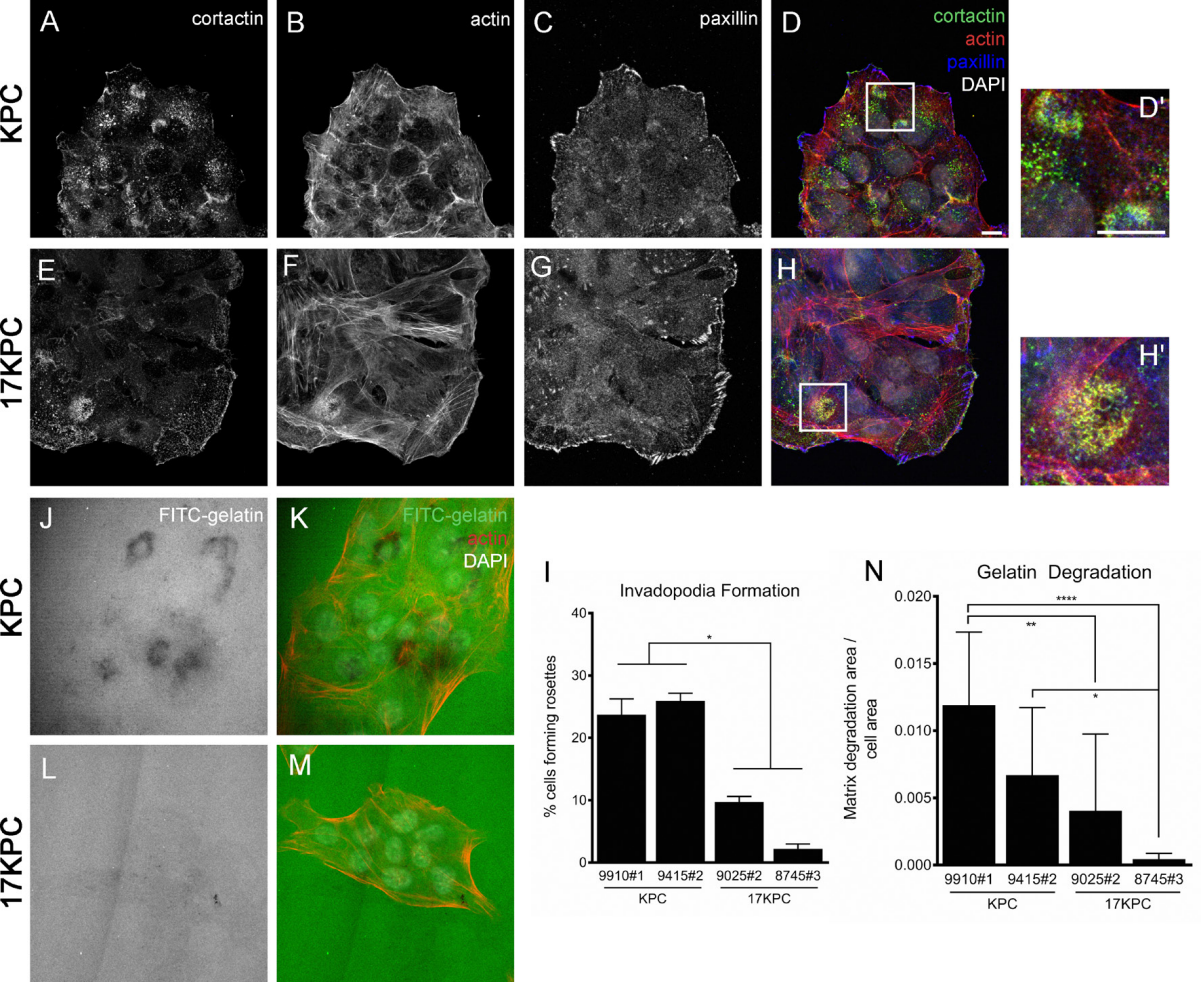
*mir-17~92* null PDAC cell lines form fewer invadopodia rosettes Immunofluorescence staining for the invadopodia constituent proteins cortactin (**A**, **E**), actin (**B**, **F**) and paxillin (**C**, **G**) in representative KPC and 17KPC cell lines. Merged images are shown in (**D**) and (**H**). D′ and H′ are higher magnification views of panels D and H. Scale bar = 10 um. Quantification of invadopodia rosettes is shown in (**I**). *n* = 3 for each cell line. Areas of FITC-gelatin degradation, identified as dark regions, are shown for representative KPC and 17KPC cell lines (**J**, **L**). (**K**, **M**) Co-staining for actin and DNA (DAPI). (**N**) Quantification of gelatin degradation. *n* = 3 for each cell line. Error bars represent standard error of the mean. *p* values: * < 0.05, ** < 0.01, **** < 0.0001.

In cancer cells, invasion activity is associated with the presence of specific matrix-degrading, cell adhesion structures called invadopodia [51, 52]. Invadopodia can be identified by the organization of their core cytoskeletal protein components–actin and cortactin–into punctate or rosette-shaped structures that are functionally associated with localized sites of elevated metalloproteinase activity [51, 53–55]. To better understand the nature of the invasive defect that we observed in 17KPC cell lines, we analyzed invadopodia formation by immunofluorescence. We found that invadopodia in KPC and 17KPC cell lines take the form of rosettes and that KPC cell lines display significantly higher rates of invadopodia rosette formation than 17KPC lines (Figure 6A–6I). In agreement with reduced invadopodia numbers, 17KPC cell lines also degrade less FITC-labeled gelatin matrix than KPC lines (Figure 6J–6N). These data suggest that loss of *mir-17~92* decreases invasion, at least in part, as a result of reduced matrix-degrading capacity.

### miR-19 promotes invadopodia formation and the invasiveness of pancreatic cancer cell lines

**Figure 7:**
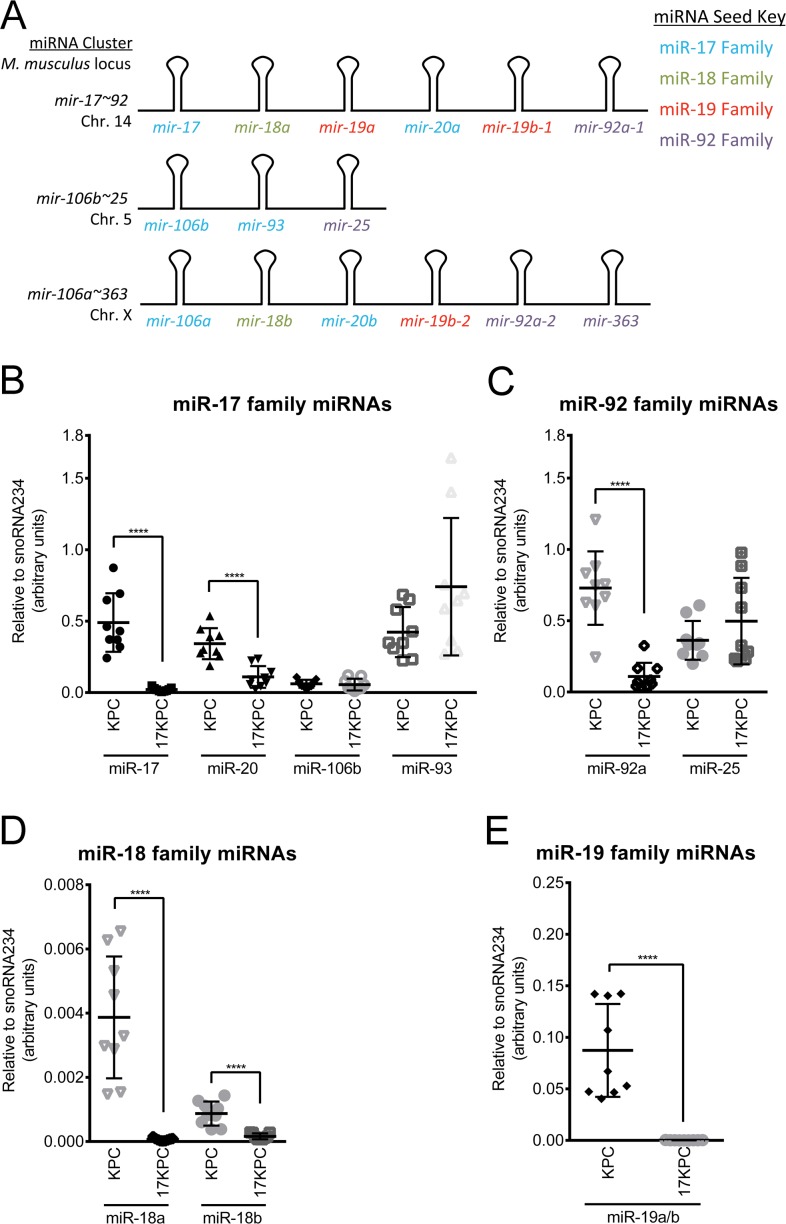
miR-19 family expression is absent in 17KPC cell lines (**A**) Schematic representation of the *mir-17~92* cluster and its paralogs *mir-106b~25* and *mir-106a~363*. Constituent miRNAs are color-coded according to their seed families. (**B**–**E**) Quantitative RT-PCR measurement of mature miRNA expression across eighteen cell lines derived from primary KPC and 17KPC tumors. Some miRNAs share sufficient sequence similarity that standard oligonucleotides amplify both species equally (e.g. miR-19a and miR-19b), and therefore not all miRNAs are individually plotted. *p* values: **** < 0.0001. Error bars represent standard deviation from the mean.

The *mir-17~92* cluster encodes six miRNAs encompassing four miRNA seed families (Figure 7A), implicating thousands of predicted mRNA targets as downstream effectors of the cluster's invasive program. To aid in our determination of which miRNA families may be most important in the invasive phenotype, we evaluated nine KPC and nine 17KPC cell lines for their expression of miR-17, -18, -19, and -92 family miRNAs across the three cluster paralogs: *mir-17~92*, *mir-106b~25*, and *mir-106a~363*. Quantitative RT-PCR demonstrated that 17KPC cell lines are indeed null for miRNAs from *mir-17~92*, however they retain robust expression from *mir-106b~25* (Figure 7B, 7C). In fact, the *mir-106b~25* locus is sufficient to drive expression of miRNAs for the miR-17 and miR-92 families to levels close to those observed in KPC lines, suggesting that loss of the miR-17 and -92 families may not be primarily responsible for the invasive defect of 17KPC cell lines. In contrast, miR-19 family miRNAs can only be expressed from the *mir-17~92* and *mir-106a~363* clusters, and 17KPC lines were found to completely lack expression of this miRNA family (Figure 7E). Based on the partial residual expression of the miR-17 and miR-92 families (Figure 7B, 7C), and the generally very low expression of the miR-18 family (Figure 7D, note y axis units), we hypothesized that loss of the miR-19 family was responsible for the defective invasion of 17KPC cell lines.

To validate that miR-19 family miRNAs play an important role in invasion, we utilized antagomirs–short oligonucleotides that bind and inactivate miRNAs–to specifically knock down miR-19 function in KPC lines with high invasive capacity and varying levels of miR-19 expression [56]. We first confirmed miR-19 antagomir activity using a β-galactosidase (β-Gal) reporter containing multiple miR-19 binding sites within the 3′UTR that allow translational suppression in the presence of miR-19. Pooled antagomirs against miR-19a and miR-19b enhanced reporter activity in a KPC cell line, but not in a 17KPC cell line (Supplementary Figure 9).

**Figure 8:**
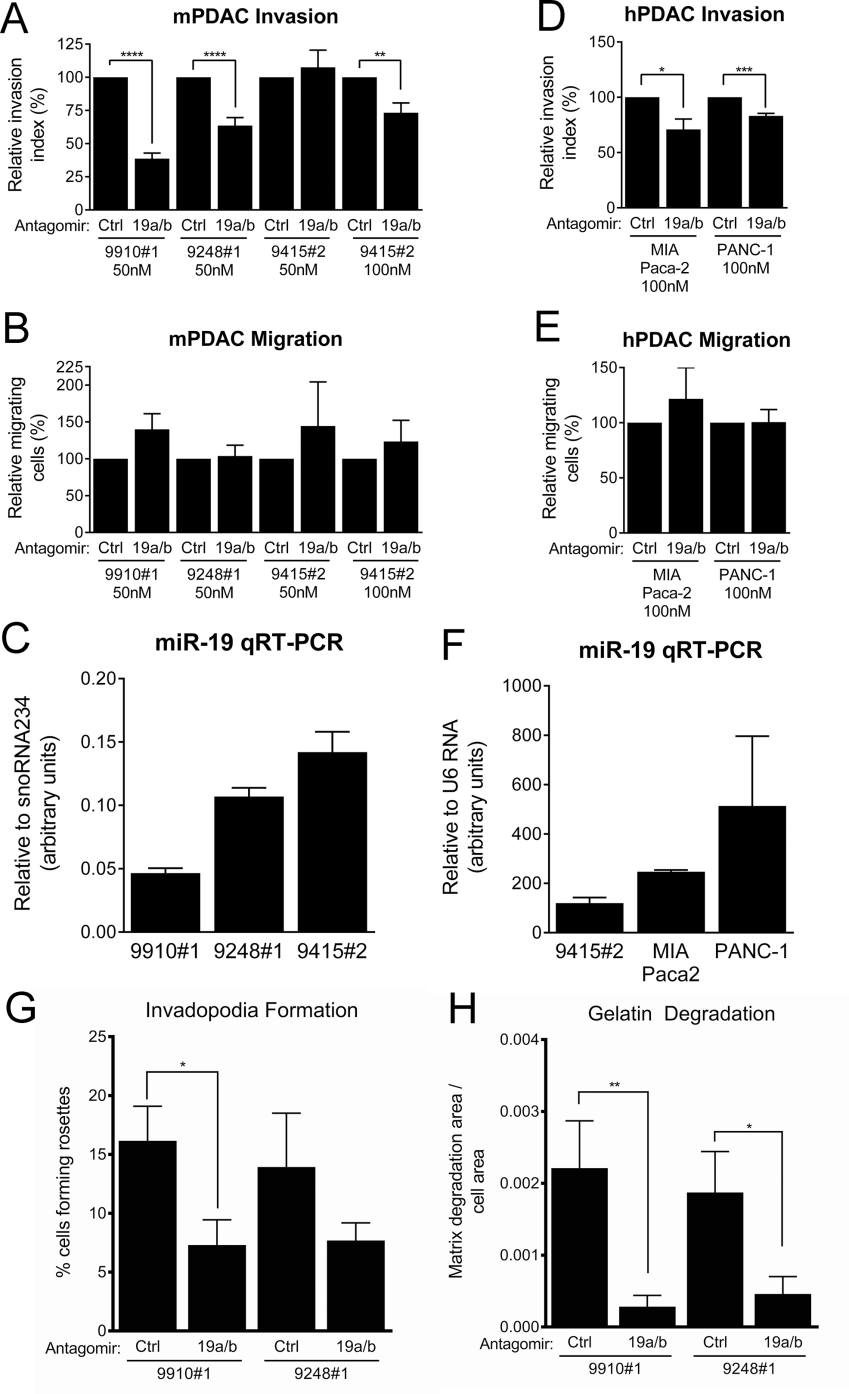
PDAC cell line invasion and invadopodia formation is suppressed by miR-19 antagomirs (**A**) Transwell invasion through Matrigel of KPC cell lines treated with control or miR-19-targeting antagomirs at the indicated concentrations. (**B**) Transwell migration activity of the cell lines shown in (A). (**C**) Quantitative RT-PCR measurement of baseline mature miR-19 levels in KPC cell lines. (**D**) Transwell invasion through Matrigel of the human PDAC cell lines MIA Paca-2 and PANC-1 treated with control or miR-19-targeting antagomirs at the indicated concentrations. (**E**) Transwell migration of activity of the cell lines shown in (D). (**F**) Quantitative RT-PCR measurement of baseline mature miR-19 levels in MIA Paca-2 and PANC-1 cells; miR-19 levels in the KPC cell line 9415#2 are shown for comparison. Invadopodia rosette formation (**G**) and FITC-gelatin degradation (**H**) in the KPC cell lines 9910#1 and 9248#1 treated with control or miR-19-targeting antagomirs. *n* = 3 for each cell line. *p* values: * < 0.05, ** < 0.01, *** < 0.001, **** < 0.0001. (A–F) Error bars represent standard deviation from the mean. (G, H) Error bars represent standard error of the mean.

MiR-19 antagomirs significantly inhibited KPC cell line invasion, but not migration, consistent with an invasion-specific effect for miR-19 family miRNAs (Figure 8A, 8B). This response inversely correlated with the level of endogenous miR-19 family expression, suggesting a dosage response (Figure 8C). Indeed, the cell line with the highest expression of miR-19, 9415#2, was resistant to antagomirs at a concentration of 50 nM, but responded when treated with antagomirs at 100 nM (Figure 8A, 8C). We next ascertained whether miR-19 regulates invasion in human PDAC cells. The human pancreatic cancer cell lines MIA Paca-2 and PANC-1 are also invasive and express relatively high levels of miR-19 (Figure 8F). Treatment of these lines with miR-19 antagomirs reduced their invasive capacity in a transwell assay without affecting their migration ability (Figure 8D, 8E). Furthermore, treatment with miR-19 antagomirs was sufficient to reduce the number of invadopodia rosettes formed in KPC cells (Figure 8G, Supplementary Figure 10) and also decreased the gelatin degradation capacity of these cell lines (Figure 8H, Supplementary Figure 11). These data demonstrate that miR-19 miRNAs regulate PDAC cell invasion.

## DISCUSSION

Given its rate of mutational activation in PDAC and past observations of oncogene addiction, KRAS and components of its downstream signaling pathways represent robust therapeutic targets in this devastating malignancy. To date, efforts to directly inhibit KRAS function have been unsuccessful [8]; thus efforts in the field have turned to targeting key downstream pathways [9–11]. However, a deeper understanding of the mechanisms responsible for the transforming effect of KRAS could inform more effective therapeutic strategies. Several microRNAs, including those in the *mir-17~92* cluster, display increased expression in PDAC as well as precursor PanIN lesions [22–24], suggesting that these miRNAs may play a role in tumorigenesis. However, to date, the functional importance of these miRNAs has not been evaluated *in vivo*.

Here we report that deletion of the *mir-17~92* miRNA cluster results in the regression of KRAS^G12D^-driven PanIN lesions and the expansion of normal acinar tissue in place of neoplastic cells over time. In addition, we observe that *mir-17~92*-null PanIN lesions have reduced ERK phosphorylation, and PanIN cell lines with ectopic *mir-17~92* expression display elevated p-ERK levels. However, *mir-17~92*-null PanIN lesions display no changes in MEK phosphorylation or PI3K/AKT signaling, suggesting a specific impact of *mir-17~92* on ERK activation. Importantly, apoptotic rates are not increased in *mir-17~92*-null PanINs, suggesting that these lesions are not lost by apoptosis. Moreover, the proliferation rate is unchanged in the adjacent acinar tissue of 17KC mice compared to that of KC mice, indicating that exocrine recovery is not due to the expansion of residual acinar cells in these animals. Together, these findings suggest that *mir-17~92* loss promotes the redifferentiation of PanINs into acinar cells. While sophisticated lineage tracing experiments will be required to validate this hypothesis, our data are in agreement with recently published data demonstrating that ERK pathway activity downstream of KRAS^G12D^ is critical for PanIN maintenance, and loss of ERK signaling triggers PanIN regression into normally differentiated exocrine tissue [45]. Importantly, the effects of miRNAs on signaling pathway output are generally smaller in magnitude than those observed with small molecule inhibitors, which potentially explains the extended timeline of PanIN regression in 17KC mice compared to the rapid regression described by Collins, *et al*. using MEK inhibitors [45].

The mechanisms underlying the regulation of ERK activity by *mir-17~92* are unknown. We explored the possibility that *mir-17~92* increases ERK activation via suppression of dual-specificity phosphatases (DUSPs), which are well-known regulators of numerous MAPK family proteins, including ERK [57]. In particular, DUSP2, DUSP7 and DUSP10 suppress ERK activity and are demonstrated or predicted targets of *mir-17~92* miRNAs [48, 58–60]. Interestingly, a recent publication linked miR-92 and DUSP10 to PDAC cell proliferation *in vitro*, suggesting that there may indeed be an important role for this regulatory axis in pancreatic tumorigenesis [61]. However, immunohistochemical staining of DUSP2, DUSP7 and DUSP10 failed to demonstrate any difference between KC and 17KC PanIN lesions, and modulation of *mir-17~92* levels in PanIN cell lines also failed to change the levels of these phosphatases. Thus, the precise mechanisms through which *mir-17~92* regulates ERK phosphorylation remain unknown. Additional studies using inducible expression or repression of *mir-17~92* miRNAs, coupled to mRNA and proteomic profiling approaches, will aid in elucidating the mechanisms by which this cluster regulates ERK phosphorylation in the early stages of pancreatic tumorigenesis.

We observed similar overall survival, rates of metastasis, and histological prevalence of invasion at sacrifice between KPC and 17KPC mice, suggesting that *Trp53* loss can compensate for *mir-17~92* deletion. In the KPC model, mice typically develop aggressive PDAC, characterized by local invasion, obstructive symptoms involving the gastrointestinal or biliary systems, and metastasis, which we observe in the KPC animals of this study. In contrast, we find that 17KPC mice exhibit less aggressive primary disease, as demonstrated by longer survival with larger primary tumors in the absence of metastases, and the absence of obstructive gastrointestinal or biliary symptoms. These data suggest that *mir-17~92* plays a role in PDAC invasiveness. However, we cannot exclude the possibility that the absence of obstructive disease in the 17KPC mice reflects a difference in the initial anatomical site of these primary tumors compared to KPC tumors. Perhaps the tumors in 17KPC mice arise in the tail of the pancreas, allowing them to reach greater size before obstructing the duodenum or bile duct; whereas tumors occurring in KPC animals predominantly occur in the head of the pancreas, predisposing those animals to early obstructive phenomena. Additional studies analyzing tumors at earlier stages and smaller sizes will be needed in order to clarify whether KPC and 17KPC tumors arise in different locations within the pancreas.

Analysis of a panel of tumor-derived cell lines demonstrated that 17KPC cells are specifically defective in their ability to form invadopodia rosettes and invade through Matrigel in a transwell assay, providing a potential link to our *in vivo* observations. Using antagomirs against miR-19, we demonstrated that miR-19 family miRNAs are key drivers of invadopodia formation and the invasive capacity of human and murine pancreatic cancer cells. Thus, we have identified a novel role for miR-19 in pancreatic cancer cells. In fact, few studies exist that link miR-19 to cancer cell invasion in any tumor type [36, 37]. Future studies will be required to validate the significance of miR-19 family miRNAs in the invasive phenotype of PDAC *in vivo*. In addition, expression profiling and proteomic studies will be required to identify the mechanisms through which miR-19 miRNAs regulate invadopodia formation and function. TIMP2, CST3, and TGM2 are all predicted targets of miR-19 that are also suppressors of invasion [37, 39, 62, 63] and could potentially explain the reduced invasiveness of 17KPC cell lines. Indeed, TGM2 is linked to miR-19-mediated invasion in colorectal cancer cells [37]. However, immunoblotting and qRT-PCR experiments failed to detect any difference in expression between KPC and 17KPC cell lines (data not shown). Matrix metalloproteinases are also key drivers of invasion [64], and a survey of MMP-2, -7, -9, and MT1-MMP by qRT-PCR demonstrated no difference between KPC and 17KPC cell lines (data not shown). Given that our data point to a major effect of miR-19 on invadopodia formation and/or stability, and since none of the above factors are known to influence invadopodia, these negative findings are perhaps not surprising. Instead, they highlight the importance of the unbiased approaches stated above for elucidating the mechanisms responsible for the observed phenotypes.

Importantly, our experiments do not preclude significant roles for the other miRNAs encoded within the *mir-17~92* cluster in PDAC invasion. Indeed, other members of the cluster are computationally predicted to regulate genes previously implicated in cellular invasion. Thus, studies that confirm or exclude roles for these miRNAs in PDAC invasion will also be of importance.

Our studies reported here are potentially in conflict with the recently published work of Heeschen and colleagues [65, 66]. In their work, the authors report that ectopic *mir-17~92* expression promotes the proliferation of pancreatic cancer stem cells, resulting in their premature depletion and consequent reduced cell transformation and tumorigenicity. In contrast, our studies demonstrate that ectopic *mir-17~92* increases MEK/ERK signaling, a feature required for the maintenance of established PanIN lesions [6, 67], suggesting that elevated *mir-17~92* levels should enhance pancreatic cancer development. However, we have not evaluated the self-renewal capacity of *mir-17~92* expressing PanIN cell lines, nor have we tested their tumorigenic capacity upon implantation into recipient mice. Future experiments in genetically engineered PDAC mouse models with enhanced *mir-17~92* expression may be required to resolve this discrepancy.

Together, our findings demonstrate important roles for *mir-17~92*-encoded miRNAs during early stages of pancreatic tumorigenesis, as well as tumor progression and invasion. These findings provide functional support for the observed elevated expression of these miRNAs in precursor PanIN lesions and invasive PDAC. Further dissection of the cluster to identify the roles played by individual miRNAs during PanIN maintenance and PDAC invasion will be required in order to identify the critical target genes and pathways regulated by this miRNA cluster during pancreatic tumorigenesis.

## MATERIALS AND METHODS

### Animal studies

The *Ptf1a-Cre* [40], *mir-17~92^flox^* [41], *LSL-Kras^G12D^* [44], and *Trp53^flox^* [68] mouse strains have been described previously. Health status of all animals was monitored at least three times per week, and animals were euthanized when they displayed signs of distress or high tumor burden. Animals were maintained in specific pathogen-free facilities with abundant food and water. Mice were randomly assigned to the studies. Group sizes were estimated based on investigators’ prior experience. Euthanized mice that did not have pancreatic tumors were censored from the analysis. Mice of both genders were used in all studies. The pathologist was blinded to mouse genotypes for quantification of histopathologic lesions. All animal experiments were reviewed and approved by the University of Massachusetts Medical School Institutional Animal Care and Use Committee.

### Histological stains

Tissues were fixed in 10% neutral-buffered formalin. Five-micrometer sections on charged glass slides were cleared through Xylenes (Fisher #X3P) to 100% ethanol and rehydrated through a graded alcohol series to distilled water. Immunostaining was performed as previously described [69]. A list of all antibodies used for immunohistochemical stains appears in Supplementary Table 2. Stains were developed using ABC (Vector Labs #PK-6101) and Nova Red (Vector Labs #SK-4800) kits according to manufacturer's instructions.

Hematoxylin and eosin stains were performed according to field standards, and the quadchrome stain (consisting of a hybrid protocol derived from Sirius Red staining for collagen and Alcian Blue staining for mucin) was performed as recently described [47].

A licensed pathologist who was blinded to tissue genotypes performed quantification of tissue areas. Four images were quantified per section representing 1) the area of greatest neoplastic progression, 2) the area of lowest neoplastic progression, and 3,4) areas of the pancreas that were consistent with the average progression for that tissue section. Acini, ducts, PanINs, ADM lesions, and stromal tissue not including blood vessels were manually outlined using ImageJ software and are graphed as the percentage area of all areas quantified.

### Cell culture experiments

All cell lines were maintained at subconfluent densities in high glucose DMEM (Life Technologies #11965) supplemented with 10% fetal bovine serum (Atlanta Biologicals #S11150) and 100U/ml penicillin/streptomycin (Pen/Strep: Life Technologies #15140) (herein ‘complete media’). Murine PDAC cell lines were generated in the Lewis lab. Murine PanIN cell lines were obtained from Nabeel Bardeesy (Massachusetts General Hospital). Human PDAC cell lines were obtained from ATCC, except for the Pa01c through Pa18C cell lines, which were obtained from Bert Vogelstein (Johns Hopkins University) [70]. Migration and invasion assays were performed as previously described using 8-micrometer-porous transwell inserts (Fisher #08-774-162) and Matrigel-coated inserts (Fisher #08-774-122) [71]. The invasion index was calculated as previously described [71]. Soft agar colony formation assays were performed as previously described [69]. Proliferation assays of adherent, subconfluent cells were performed by direct live cell counting after trypsin-mediated resuspension using trypan blue exclusion over a period of 48 hours. Counts were plotted as the log_2_ of the cell number over time, and the proliferative rate of each line was calculated as the inverse slope of a linear regression to the data (i.e. time/doubling). Data shown are the average of greater than four experiments. *P*-values were calculated using the Student's *t-test*.

For antagomir experiments, miRCURY LNA Power Inhibitors (Exiqon #4101004-100, #4103258-100, and #199006-100) were transfected into 2.5 × 10^5^ cells per well of a 6-well plate using Superfect (Qiagen #301305) according to manufacturer's specifications at a final antagomir concentration of either 50 or 100 nM.

Lentiviral infection of the human PanIN cell lines RP2294 and AH2375 was performed using viral vectors generously provided by Andrea Ventura, PhD [34].

### Invadopodia and gelatin degradation analysis

Invadopodia rosette formation analysis and FITC-gelatin matrix degradation assays were performed as previously described [72]. Briefly, to visualize invadopodia rosette formation, cells were plated on fibronectin-coated slips (10μg/ml; Corning) for 24hrs, fixed, permeabilized and stained with anti-cortactin antibodies (1:200; Merck Millipore #05-180), anti-paxillin antibodies (1:200; Santa Cruz Biotechnology #sc-5574), TRITC-phalloidin (F-actin)(1:1000; Invitrogen #R415) and DAPI (nuclei) (1:1000; Sigma-Aldrich #D9542). A minimum of 150 cells per sample were scored for rosette formation, which were defined by cortactin and actin colocalization. *n* = 3.

For FITC-gelatin degradation analysis, coverslips were coated with 50μg/ml poly-L-lysine (Sigma-Aldrich #P8920) in PBS, and then incubated with 0.5% glutaraldehyde (Sigma-Aldrich #G6257) in PBS. The slips were coated with 1:40 fluorescent 488 gelatin (‘FITC-gelatin’) (Life Technologies #G13186) diluted with 0.2% unlabeled gelatin (Sigma-Aldrich) in PBS for 30 min. at 37°C. Cells were plated for 24 hours, fixed and stained as above. For quantitation of matrix degradation, nine random fields (20x objective) were imaged from each sample (*n* = 3). ImageJ software was used to threshold the cell area as well as areas of matrix degradation (black areas in FITC-gelatin) and to calculate a ratio of matrix degradation to cell area. Statistical analyses were performed using a one-way ANOVA with a Tukey correction.

### qRT-PCR

Quantitative RT-PCR for miRNAs was performed as previously described [73]. After RNA isolation with TRIzol reagent (Invitrogen #15596), genomic contaminants were removed using DNAse (Life Technologies #AM1907). DNA-free RNA was then polyadenylated (New England Biolabs #M0276) and subsequently reverse transcribed (Invitrogen #18080) using a pool of specially designed primers at a concentration of 50 uM to generate cDNA copies of polyadenylated miRNAs. A complete list of primer sequences appears in Supplementary Table 3. All subsequent steps of the cDNA synthesis were conducted according to kit directions.

PCR for miRNAs was performed as follows: 1) denaturation at 94°C for 15 seconds, 2) annealing at 55°C for 30 seconds, and 3) extension at 70°C for 34 seconds, for a total of 40 cycles. The reverse primer for all miRNA reactions was the sequence of the universal tag present in all 12 RT primers. The forward primer for each miRNA was the mature miRNA sequence given by miRBase [74]. PCR reactions were carried out on an ABI Step One Plus machine in 10ul volumes using SYBR Green (VWR #95072).

C_T_ values were calculated for all miRNA PCR reactions at a uniform threshold of absorbance across all experiments and controlled to C_T_ values for the endogenous reference (snoRNA234 in mouse-only experiments, U6 in human and cross-species comparisons). The expression of individual miRNAs is presented as relative snoRNA234 units in order to convert the ΔC_T_ value for each miRNA into a relative molar measure (calculated as 2^ΔCT^).

## SUPPLEMENTARY MATERIALS TABLES AND FIGURES



## Abbreviations

PDAC: pancreatic ductal adenocarcinoma
PanIN: pancreatic intraepithelial neoplasia
ERK: extracellular regulated mitogen activated protein kinase
MEK: mitogen activated protein kinase kinase
PI3K: phosphatidylinositol 3-kinase
