# Supplementary material for: MicroRNAs of the mir-17~92 cluster regulate multiple aspects of pancreatic tumor development and progression
Source: Oncotarget. 2017 Mar 16;8(22):35902–18. doi: 10.18632/oncotarget.16277 (PMC5482626; doi:10.18632/oncotarget.16277)
Supplement: Supplementary file 1 [file oncotarget-08-35902-s001.pdf]

MicroRNAs of the *mir-17~92* cluster regulate multiple aspects of pancreatic tumor development and progression

Supplementary Materials

Supplementary Table 1: Pathological characterization of KPC and 17KPC tumors

Case #	Genotype	Pred. Invasive	Other Invasive	% Invasive	Structures Invaded
8657	17KPC	Tub	Sarc	75	n/a
8745 #3	17KPC	Sarc	Tub	60	n/a
8849 #3	17KPC	Sarc	Tub	90	Duod ME
8860 #2	17KPC	Sarc	Tub	60	fSt ME, LN, Duod SM
8964 #1	17KPC	Tub	Sarc	30	LN
8964 #3	17KPC	Tub	Sarc	40	LN
8965 #2	17KPC	Sarc	Tub	60	Duod SM
8965 #3	17KPC	Tub	Sarc	70	LN, Colon Muc
9025 #2	17KPC	Sarc	Tub	70	Stom SM
9096 #1	17KPC	Sarc	Tub	70	Kid Pelvis, Duod SM
9096 #2	17KPC	Sarc	Tub	40	n/a
9158 #4	17KPC	Sarc	Tub	30	Duod ME
9327 #3	17KPC	Sarc	Tub	70	n/a
9486 #3	17KPC	Sarc	Tub	70	n/a
9975 #1	17KPC	Sarc	n/a	40	Duod ME
9976 #1	17KPC	Sarc	Tub	50	Duod Muc, LN
9237 #1	KPC	Tub	Sarc	60	Duod Muc
9248 #1	KPC	Tub	Sarc/Cyst	70	LN , fSt ME
9249 #2	KPC	Sarc	Tub	70	Duod SM
9414 #3	KPC	Sarc	n/a	10	n/a
9415 #2	KPC	Sarc	Tub	60	Liver
9529 #1	KPC	Sarc	n/a	90	LN, Vein
9530 #1	KPC	Sarc	Tub	60	Liver, Vein
9550 #2	KPC	Tub	Sarc	< 10	n/a
9551 #4	KPC	Tub	Sarc	20	Duod Muc
9619 #5	KPC	Sarc	Tub	60	Duod Muc
9666 #1	KPC	Tub	Sarc	60	LN, fSt ME
9739 #3	KPC	Sarc	Tub	80	Colon ME
9762 #3	KPC	Sarc	n/a	< 10	n/a
9846 #1	KPC	Sarc	Tub	10	n/a
9909 #3	KPC	Sarc	Tub	90	n/a
9910 #1	KPC	Sarc	Tub	30	n/a
9910 #2	KPC	Sarc	Tub	70	n/a

Representative sections of each tumor were analyzed by a licensed pathologist to determine the predominant and subsidiary histological phenotypes of invasive carcinoma, as well as the percentage of the tumor area that was invasive. Anatomical structures invaded by the tumor are also noted. Tub = tubular, Sarc = sarcomatoid, Cyst = Cystic, Duod = duodenum, LN = lymph node, Stom = stomach, fSt = forestomach, Kid = kidney, ME = muscularis externa, SM = submucosa, Muc = Mucosa.

Supplementary Table 2: Antibodies and conditions

	Stain	Cat. Number	Antibody Concentration	Time	Temp
Co-immunofluorescence	anti-Insulin	Dako A0564	1:500	4 hr	RT
	anti-Glucagon	Dako A0565	1:50	4 hr	RT
	Alexa-fluor-594 anti-rabbit	ab150080	1:250	1 hr	RT
	FITC anti-guinea	Jackson Immuno: 706-095-148	1:500	1 hr	RT
	anti-Cortactin	Millipore 05-180	1:200	1 hr	RT
	anti-Paxillin	SC-5574	1:200	1 hr	RT
	TRITC-phalloidin	Invitrogen #R415	1:1000	1 hr	RT
Immunohistochemistry	anti-ki67	ab66155	1:600	4 hr	RT
	anti-CC3	cs-9664	1:800	4 hr	RT
	anti-pAKT^{T308}	CST-4056	1:200	1 hr	RT
	anti-pAKT^{S473}	CST-4060	1:400	O/N	4°C
	anti-pERK	CST-9101	1:400	1 hr	RT
	anti-pMEK 1/2	SC-7995	1:1000	1 hr	RT
Immunoblotting	anti-α-tubulin	DSHB 12G10	1:1000	O/N	4°C
	anti-pERK	CST 9101	1:2000	O/N	4°C
	anti-total ERK	CST 9102	1:1000	O/N	4°C
	anti-DUSP7	PA5-15553	1:1000	O/N	4°C
	anti-DUSP10	ab140123	1:2000	O/N	4°C

Blocking were as follows: immunohistochemistry: 10% normal goat serum in PBS overnight at 4°C; immunoblotting: 5% milk in TBS-T except for anti-DUSP7 which was blocked in 5% BSA. RT: room temperature; O/N: overnight.

Supplementary Table 3: Primers used in PCR reactions

Primer Name	Primer Sequence (5′-3′)
miR-17	CAAAGTGCTTACAGTGCAGGTAG
miR-106a	CAAAGTGCTAACAGTGCAGGTAG
miR-106b-5p	TAAAGTGCTGACAGTGCAGAT
miR-20a	TAAAGTGCTTATAGTGCAGGTAG
miR-20b	CAAAGTGCTCATAGTGCAGGTAG
miR-93	CAAAGTGCTGTTCGTGCAGGTAG
miR-19a/b	TGTGCAAATCTATGCAAAACTGA
miR-18a	TAAGGTGCATCTAGTGCAGATAG
miR-18b	TAAGGTGCATCTAGTGCTGTTAG
miR-92	TATTGCACTTGTCCCGGCCTG
miR-25	CATTGCACTTGTCTCGGTCTGA
miR-363	AATTGCACGGTATCCATCTGTA
snoRNA234	GATTTAACAAAAATTCGTCACTACCACTGAGA
U6 snRNA (mmu/hsa)	CATCTCGAGCTAATCTGGTGGG
RT Primer 1	ACGCATCTATGCGCATATCG TTTTTTTTTTTTTTT AA
RT Primer 2	ACGCATCTATGCGCATATCG TTTTTTTTTTTTTTT AC
RT Primer 3	ACGCATCTATGCGCATATCG TTTTTTTTTTTTTTT AG
RT Primer 4	ACGCATCTATGCGCATATCG TTTTTTTTTTTTTTT AT
RT Primer 5	ACGCATCTATGCGCATATCG TTTTTTTTTTTTTTT CA
RT Primer 6	ACGCATCTATGCGCATATCG TTTTTTTTTTTTTTT CC
RT Primer 7	ACGCATCTATGCGCATATCG TTTTTTTTTTTTTTT CG
RT Primer 8	ACGCATCTATGCGCATATCG TTTTTTTTTTTTTTT CT
RT Primer 9	ACGCATCTATGCGCATATCG TTTTTTTTTTTTTTT GA
RT Primer 10	ACGCATCTATGCGCATATCG TTTTTTTTTTTTTTT GC
RT Primer 11	ACGCATCTATGCGCATATCG TTTTTTTTTTTTTTT GG
RT Primer 12	ACGCATCTATGCGCATATCG TTTTTTTTTTTTTTT GT
Universal Reverse Primer	ACGCATCTATGCGCATATCG
DUSP7 Fow	GACATGTTGCTAAGCGAGCG
DUSP7 Rev	TCCAGCGAGCTCGGATTTAC
DUSP10 Fow	GACCCTCAAGGCTGCGAATC
DUSP10 Rev	GCTGCATCCGCAATTCAGG

Primer sequences are provided for all qPCR primers used in the study. miRNA primers were derived according to the sequence of the mature mouse miRNA based on miRBase records. The primer for U6 snRNA is complementary to both human and mouse U6 RNA.

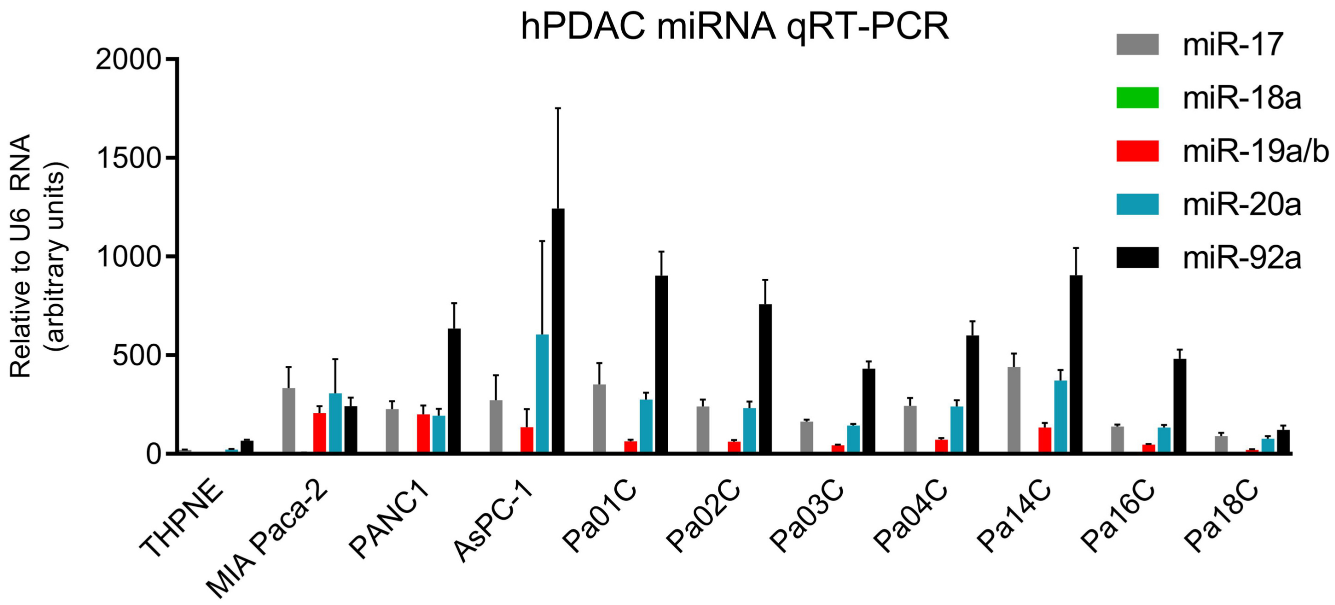

Supplementary Figure 1: Human PDAC cell lines overexpress *mir-17~92*. qRT-PCR for members of the *mir-17~92* cluster in human PDAC cell lines. miR-18 is poorly expressed compared to other members of the cluster and is not readily visualized on this scale. Error bars represent standard deviation from the mean.

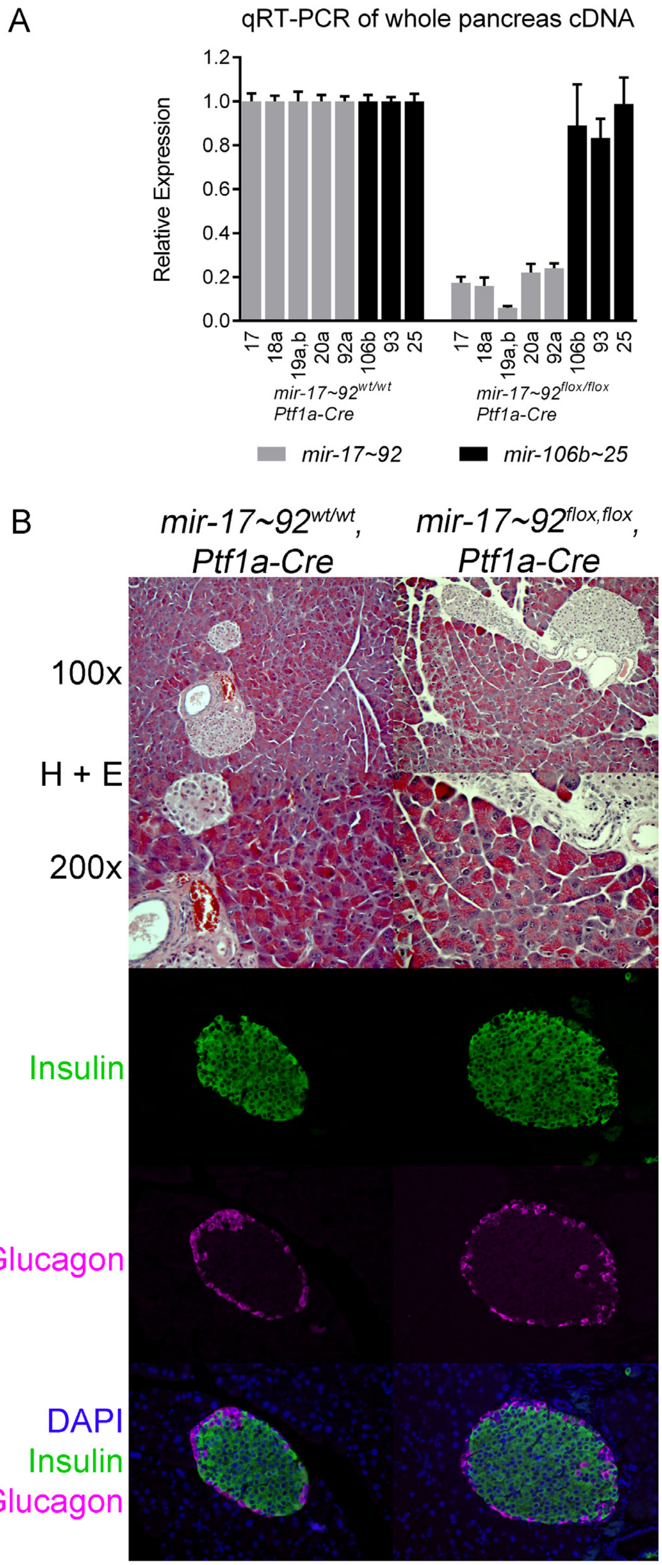

Supplementary Figure 2: Homozygous loss of *mir-17~92* in the pancreas is developmentally tolerated. (**A**) qRT-PCR measurement of levels of constituent *mir-17~92* miRNAs in pancreata from *mir-17~92$^{flox/flox}$*, *Ptf1a-Cre* animals and control littermates. (**B**) Hematoxylin and eosin (H+E) staining of pancreata from *mir-17~92$^{flox/flox}$* , *Ptf1a-Cre* animals and control littermates (top four panels). Immunofluorescence staining for the endocrine markers insulin and glucagon (lower six panels). Error bars represent standard deviation from the mean.

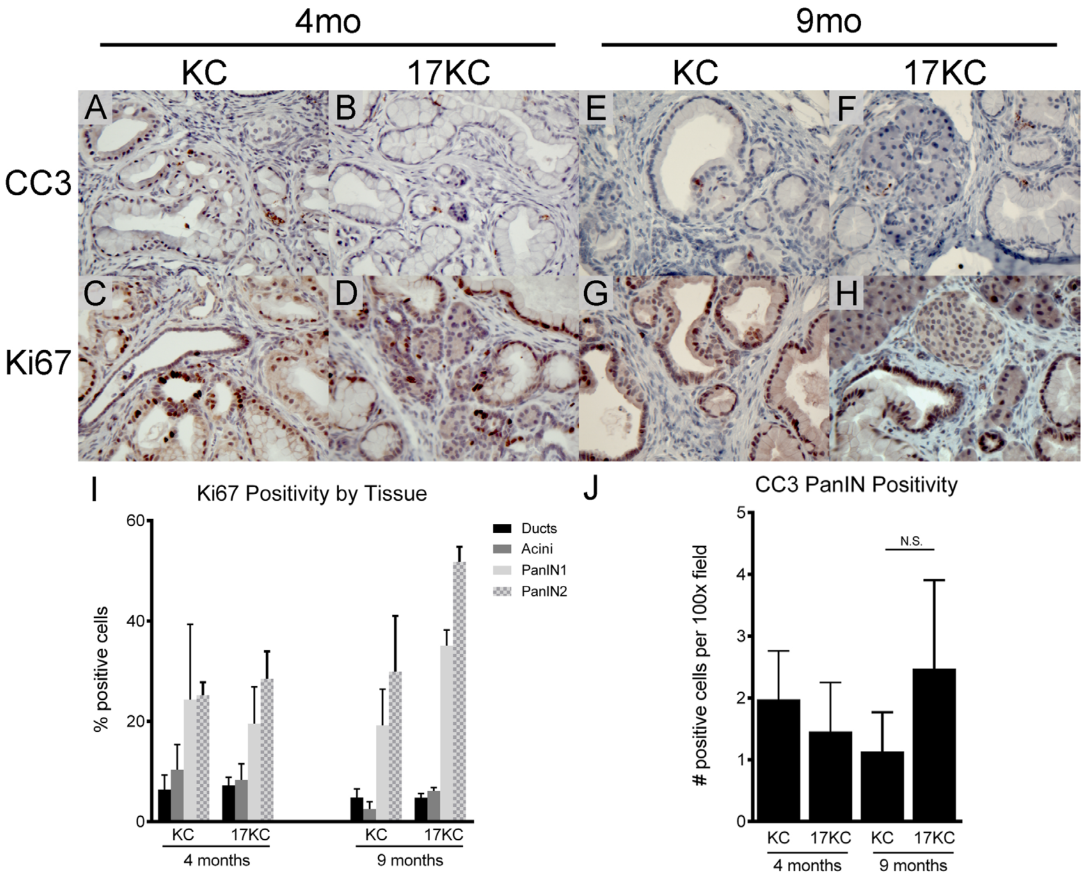

Supplementary Figure 3: KC and 17KC PanINs display similar proliferation and apoptosis rates. Immunohistochemistry for the apoptosis marker cleaved caspase 3 ('CC3'; **A**, **B**, **E**, **F**) and the proliferative marker Ki67 (**C**, **D**, **G**, **H**) in PanIN lesions from KC and 17KC pancreata. (**I**) Quantification of Ki67-positive nuclei. (**J**) Quantification of CC3-positive cells. Error bars represent standard deviation from the mean.

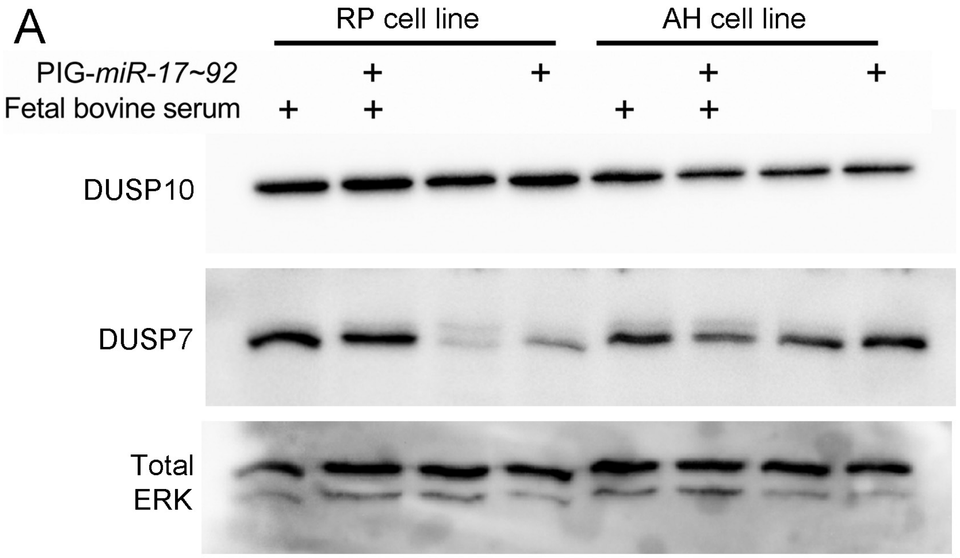

Supplementary Figure 4: *mir-17~92* overexpression in PanIN cell lines is not associated with changes in DUSP expression. Representative immunoblots for DUSP7 and DUSP10 in the PanIN cell lines RP2294 and AH2375 stably infected with PIG-*mir-17~92* or empty vector and grown under serum-free or serum replete conditions. Total ERK is used as a loading control.

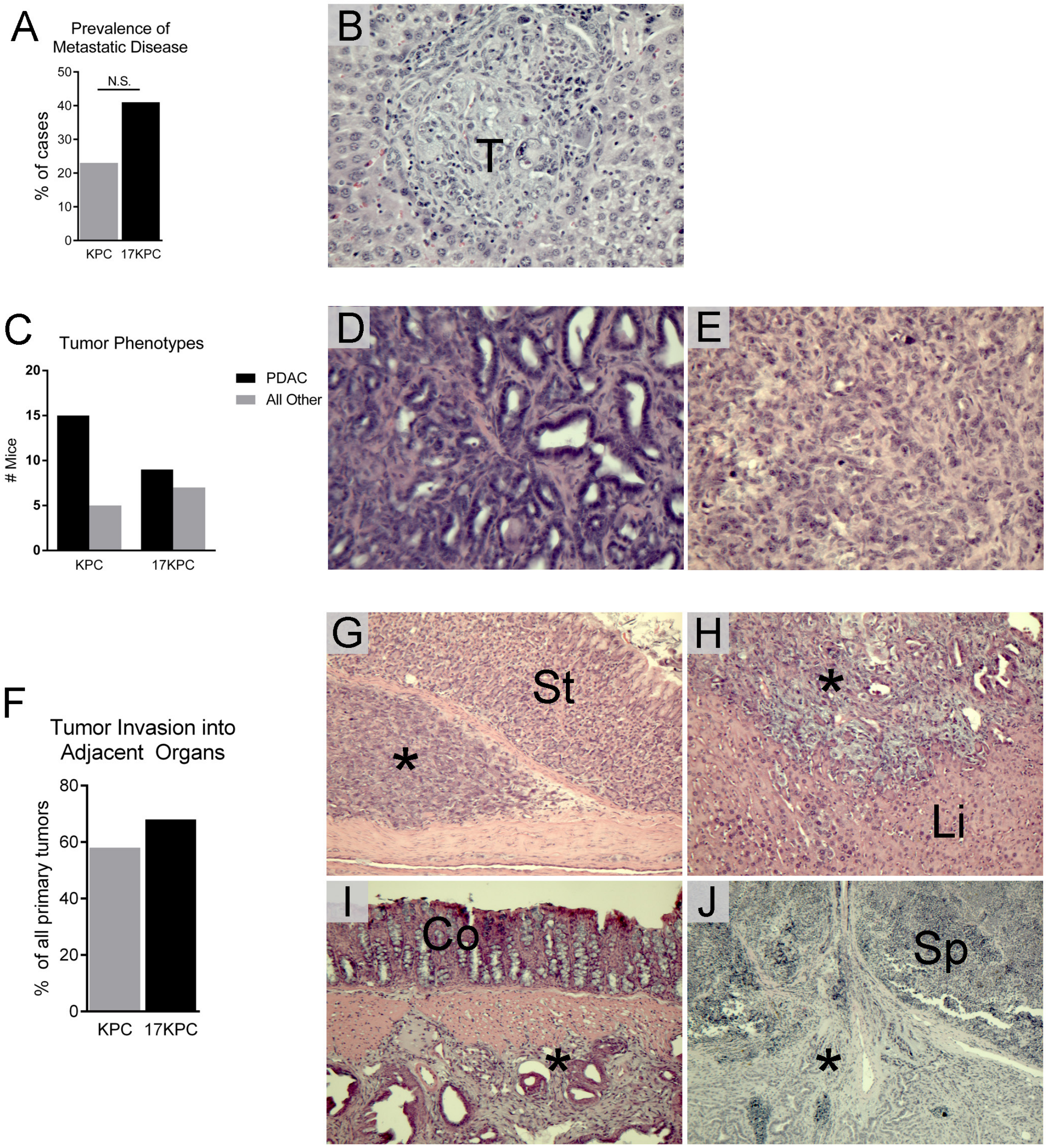

Supplementary Figure 5: Loss of *mir-17~92* does not alter disease progression among unstratified individuals. (**A**) Frequency of liver metastasis in KPC and 17KPC animals. (**B**) H&E stained section of a liver metastasis (denoted by T). (**C**) Frequency of ductal adenocarcinoma or other histology types in KPC and 17KPC mice. Examples of ductal adenocarcinoma (**D**) and poorly differentiated carcinoma (**E**) are provided. (**F**) Frequency of local invasion into adjacent structures within the abdomen by primary pancreatic tumors found in KPC and 17KPC mice. Tumors were capable of invading all portions of the stomach (**G**), liver (**H**), colon (**I**), and spleen (**J**). * denotes invading carcinoma. Li = liver, St = stomach mucosa, Sp = spleen, Co = colon mucosa.

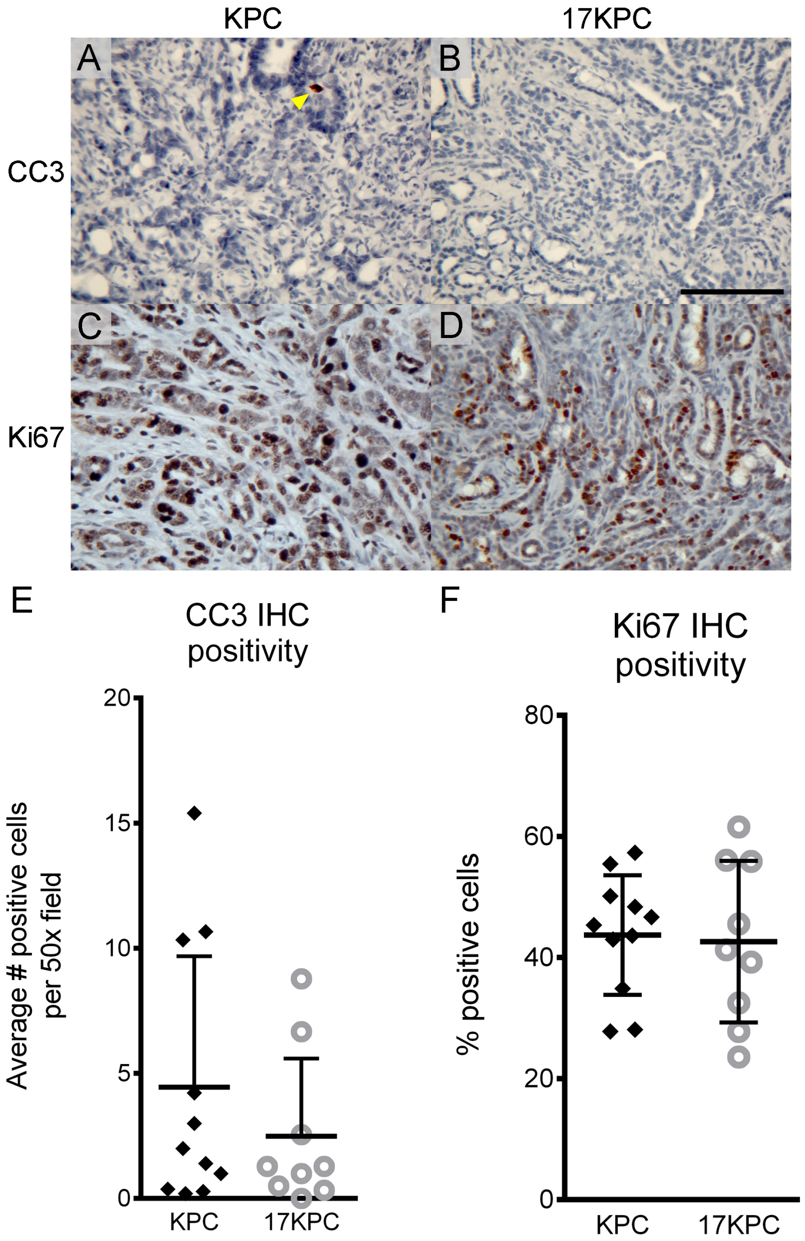

Supplementary Figure 6: Immunohistochemical staining of tumors demonstrates equivalent spectra of proliferative and apoptotic rates. (**A**, **B**) Immunohistochemical staining of KPC and 17KPC tumors for the apoptosis marker cleaved caspase 3 (CC3). (**C**, **D**) Immunohistochemical staining of KPC and 17KPC tumors for the proliferation marker Ki67. (**E**, **F**) Quantification of CC3 and Ki67 staining shown in A-D. Scale bar = 0.1mm. Yellow arrowhead in (A) indicates a rare CC3-positive nucleus. Error bars represent standard deviation from the mean.

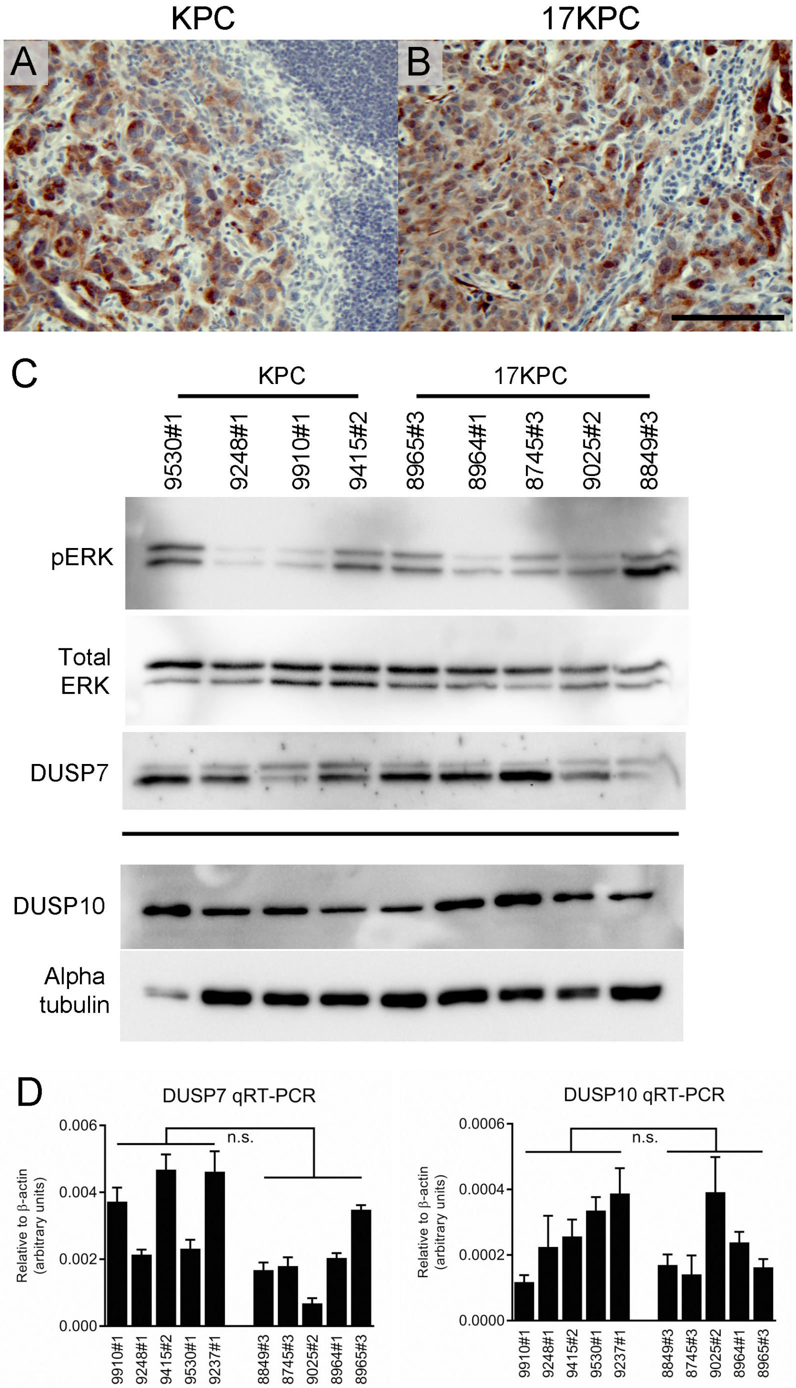

Supplementary Figure 7: ERK activity does not differ between KPC and 17KPC tumors. Immunohistochemical staining of KPC (**A**) and 17KPC (**B**) tumors for phosphorylated ERK. (**C**) Western blotting for pERK, DUSP7, and DUSP10 in KPC and 17KPC cell lines. Total ERK serves as loading control for the p-ERK and DUSP7 blots. Alpha tubulin is used as a loading control for the DUSP10 blot. (**D**) qRT-PCR for DUSP7 and DUSP10 mRNA levels in KPC and 17KPC cell lines. Scale bar = 0.1mm. Error bars represent standard deviation from the mean.

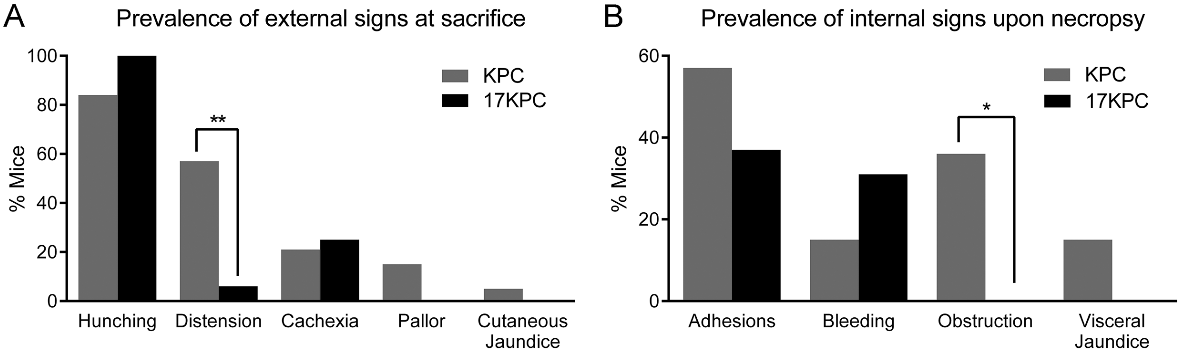

Supplementary Figure 8: 17KPC mice do not develop gastrointestinal obstruction. (**A**) Prevalence of externally visible signs of disease at time of euthanasia in KPC and 17KPC mice. (**B**) Prevalence of specific internal features noted at necropsy in KPC and 17KPC mice (notably one mouse in each cohort exhibited gross ascites upon necropsy; data not shown). *p* values: * < 0.05, ** < 0.01 by Fisher's exact test.

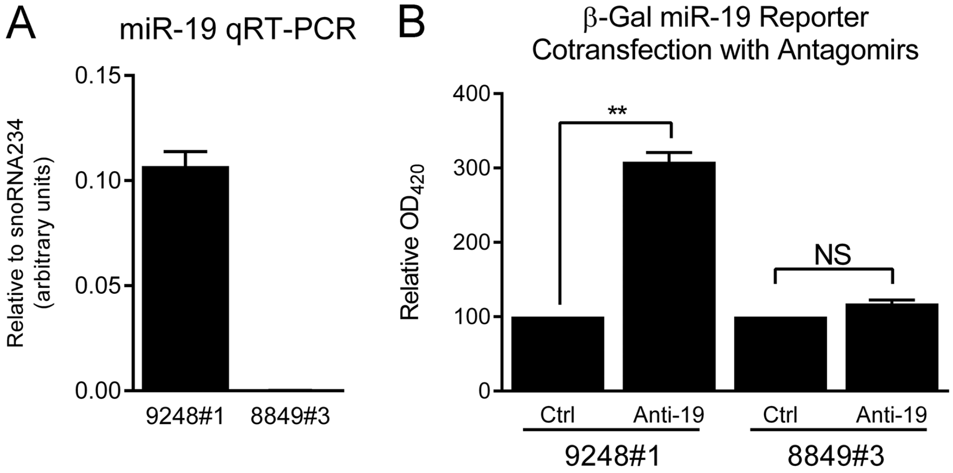

Supplementary Figure 9: miR-19 antagomirs inhibit the activity of endogenous miR-19. (**A**) Quantitative RT-PCR showing miR-19 levels in the KPC cell line 9248#1 and the 17KPC cell line 8849#3. (**B**) miR-19-responsive β-galactosidase reporter activity in the 9248#1 KPC cell line transfected with a control oligonucleotide or miR-19 antagomirs. miR-19-deficient 8849#3 cells are used as negative control. Relative density of the colorimetric reaction is plotted with control antagomir-treated samples set to 100. *p* values: ** < 0.01. Error bars represent standard deviation from the mean.

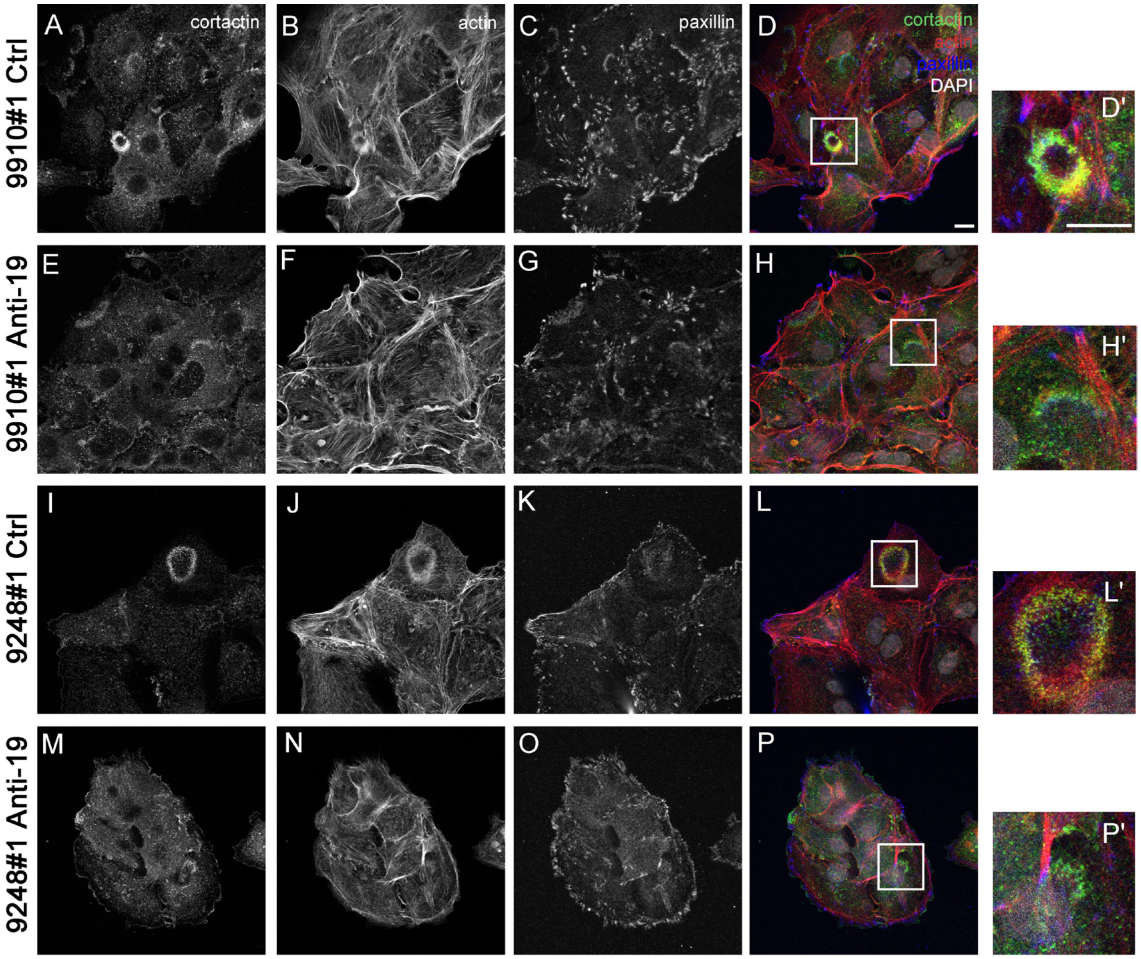

Supplementary Figure 10: miR-19 antagomirs reduce invadopodia rosette formation in KPC cell lines. Immunofluorescent staining for the invadopodia constituent proteins cortactin (**A**, **E**, **I**, **M**), actin (**B**, **F**, **J**, **N**) and paxillin (**C**, **G**, **K**, **O**) in KPC cell lines treated with either control. oligonucleotides (**A–D, I–L**) or miR-19 targeting antagomirs (**E–H, M–P**). Merged fluorescent images are shown in (**D**, **H**, **L**) and (**P**). D́, H́, Ĺ and Ṕ are L (prime) and P (prime) higher magnification views of panels D, H, L, and P. Scale bar = 10 um.

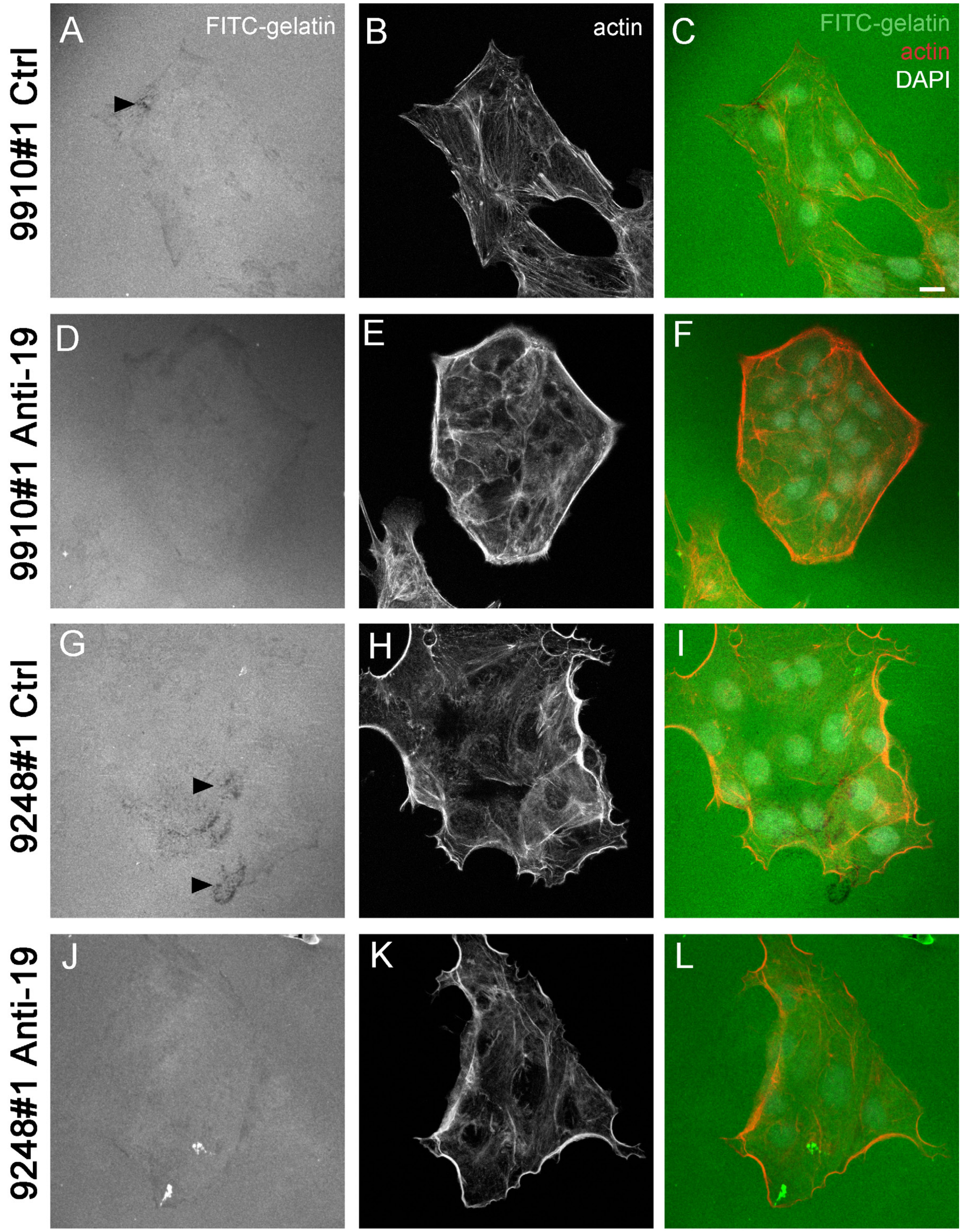

Supplementary Figure 11: miR-19 antagomirs reduce gelatin degradation in KPC cell lines. Areas of FITC-gelatin degradation, identified as dark regions (arrowheads), are shown for KPC cell lines treated with control oligonucleotides (**A**–**C**, **G**–**I**) or miR-19 antagomirs (**D**–**F**, **J**–**L**). Fluorescent staining for actin is used to identify cells on the FITC-gelatin (**B, E, H, K**). Merged images showing gelatin degradation, actin, and DAPI-stained nuclei are provided (**C, F, I, L**). Scale bar = 10 um.
